# Loss of DNMT1o Disrupts Imprinted X Chromosome Inactivation and Accentuates Placental Defects in Females

**DOI:** 10.1371/journal.pgen.1003873

**Published:** 2013-11-21

**Authors:** Serge McGraw, Christopher C. Oakes, Josée Martel, M. Cecilia Cirio, Pauline de Zeeuw, Winifred Mak, Christoph Plass, Marisa S. Bartolomei, J. Richard Chaillet, Jacquetta M. Trasler

**Affiliations:** 1 Departments of Pharmacology & Therapeutics, Pediatrics and Human Genetics, Research Institute at The Montreal Children's Hospital of the McGill University Health Centre, McGill University, Montreal, Quebec, Canada; 2 Department of Epigenomics and Cancer Risk Factors, The German Cancer Research Center, Heidelberg, Baden-Württemberg, Germany; 3 Department of Microbiology and Molecular Genetics, University of Pittsburgh, Pittsburgh, Pennsylvania, United States of America; 4 Department of Cell and Developmental Biology, University of Pennsylvania Perelman School of Medicine, Philadelphia, Pennsylvania, United States of America; Massachusetts General Hospital, Howard Hughes Medical Institute, United States of America

## Abstract

The maintenance of key germline derived DNA methylation patterns during preimplantation development depends on stores of DNA cytosine methyltransferase-1o (DNMT1o) provided by the oocyte. *Dnmt1o^mat−/−^* mouse embryos born to *Dnmt1^Δ1o/Δ1o^* female mice lack DNMT1o protein and have disrupted genomic imprinting and associated phenotypic abnormalities. Here, we describe additional female-specific morphological abnormalities and DNA hypomethylation defects outside imprinted loci, restricted to extraembryonic tissue. Compared to male offspring, the placentae of female offspring of *Dnmt1^Δ1o/Δ1o^* mothers displayed a higher incidence of genic and intergenic hypomethylation and more frequent and extreme placental dysmorphology. The majority of the affected loci were concentrated on the X chromosome and associated with aberrant biallelic expression, indicating that imprinted X-inactivation was perturbed. Hypomethylation of a key regulatory region of *Xite* within the X-inactivation center was present in female blastocysts shortly after the absence of methylation maintenance by DNMT1o at the 8-cell stage. The female preponderance of placental DNA hypomethylation associated with maternal DNMT1o deficiency provides evidence of additional roles beyond the maintenance of genomic imprints for DNA methylation events in the preimplantation embryo, including a role in imprinted X chromosome inactivation.

## Author Summary

During oocyte growth and maturation, vital proteins and enzymes are produced to ensure that, when fertilized, a healthy embryo will arise. When this natural process is interrupted, one or more of these essential elements can fail to be produced thus compromising the health of the future embryo. We are using a mouse model, lacking an enzyme (DNMT1o) produced in the oocyte and only required post-fertilization in the early embryo for the maintenance of inherited DNA methylation marks. Here, we reveal that oocytes lacking DNMT1o, when fertilized, generated conceptuses with a wide variety of placental abnormalities. These placental abnormalities were more frequent and severe in females, and showed specific genomic regions constantly deprived of their normal methylation marks. The affected genomic regions were concentrated on the X chromosome. Interestingly, we also found that a region important for the regulation of the X chromosome inactivation process was hypomethylated in female blastocysts and was associated with sex-specific abnormalities in the placenta, relaxation of imprinted X chromosome inactivation, and disruption of DNA methylation later in development. Our findings provide a novel unanticipated role for DNA methylation events taking place within the first few days of life specifically in female preimplantation embryos.

## Introduction

Genomic methylation patterns are initially differentially acquired during male and female gametogenesis by the action of the DNMT3a cytosine methyltransferase and its accessory protein DNMT3L [1]–[3]. Germline derived DNA methylation patterns are subsequently extensively reprogrammed during preimplantation development, and while methylation is lost at many sites across the genome, it is maintained on imprinted genes [4]. Two forms of DNMT1, DNMT1s and DNMT1o, maintain DNA methylation until the blastocyst stage. DNMT1s, the full length M_r_ 190,000 form of DNMT1, is expressed at all cleavage stages of preimplantation development, and appears to maintain DNA methylation at all stages except the 8-cell stage [5]–[8]. DNMT1o is missing the amino-terminal-most 118 amino acids of DNMT1s and is the M_r_ 175,000 oocyte-derived version of DNMT1 that is only expressed in oocytes and preimplantation embryos. DNMT1o protein is present only in the cytoplasm throughout early embryo development with the exception of the 8-cell stage where it is found also in all eight nuclei [8]–[13]. Although oocytes of *Dnmt1^Δ1o/Δ1o^* female mice are devoid of DNMT1o protein, they establish normal maternal genomic imprints during oogenesis through the action of DNMT3a enzymes. Once fertilized however, the resulting preimplantation embryos fail to maintain methylation patterns at the differentially methylated domains (DMDs) of imprinted loci [12]. Mouse embryos born to *Dnmt1^Δ1o/Δ1o^* female mice lose methylation on imprinted genes between the 8-cell and 16-cell stages and most embryos subsequently die before birth [5], [12], [14]. Embryonic methylation at loci other than imprinted genes, including repeat sequences, was unaffected by the DNMT1o deficiency.

The embryos of *Dnmt1^Δ1o/Δ1o^* female mice have a variety of structural abnormalities [14]. The basis of the variation has been attributed to the failure to maintain complete DMD methylation of imprinted genes at a single stage of preimplantation development [12], [14]. Loss of methylation on half of the normally methylated alleles of imprinted genes at the 8-cell stage is followed by cell divisions whereby methylation is maintained presumably by embryo-derived DNMT1s. The presence of DMDs for imprinted genes on many autosomes coupled with random assortment of sister chromatids was predicted to result in over 4000 epigenotypes, and provide an explanation for the highly variable phenotypes [5], [14]. Molecular studies showing variations in the methylation of imprinted genes in the embryos and placentae of *Dnmt1^Δ1o/Δ1o^* female mice provided further evidence of diverse epigenotypes underlying the phenotypic abnormalities [5], [14].

The timing of preimplantation maintenance methylation and DNMT1o activity in particular coincides with key X chromosome inactivation (XCI) events. In the female zygote, both X chromosomes appear active, but soon thereafter a series of X-inactivation events ensues that results in inactivation of the paternal X chromosome. Inactivation of the paternal X chromosome is first evident at the 2- to 4-cell stage and this parent-specific or imprinted X-inactivation is associated with *Xist* expression only from the paternal X (Xp) [15]–[17]. *Xist* in turn recruits various chromatin modifying complexes that eventually render the X chromosome transcriptionally inactive [18]. It has also been suggested that imprinted XCI occurs in a two-step manner, with Xp repeat elements first silenced at the 2-cell stage followed by Xp genic silencing emerging only at the 8- to 16-cell stage embryo [19]. Imprinted X chromosome inactivation or silencing of genes on the paternal X chromosome in females is complete by the blastocyst stage. Interestingly, inactivation of Xp is maintained in the extraembryonic tissues, yet reversed in the inner cell mass of blastocyst, evident as biallelic X-linked gene expression that later evolves into mosaic monoalleic expression via random XCI [16], [17].

In extraembryonic tissues, imprinted XCI is specifically observed in cells of the trophoblast lineage and in primitive endoderm-derived cells of the visceral and parietal yolk sac [20]. At the molecular level, *Xist* expression during the initiation and maintenance of random XCI is known to be controlled by an array of both cis- and trans-acting factors. Key cis-acting negative regulators of *Xist* are *Tsix* and *Xite*, a cluster harboring multiple regulatory elements and a specific enhancer of *Tsix* [21]–[24]. In contrast to random XCI, the precise nature and timing of the events involved in the initiation and maintenance of imprinted XCI have been difficult to define, primarily because of technical difficulties of working with different stages of preimplantation embryo development. In a recent study Ohhata, et al. overcame some of the technical obstacles by devising an inducible *Tsix* expression system to over-express *Tsix* during preimplantation development [25]. The induction of *Tsix* led to the repression of *Xist* associated with an increased methylation of the *Xist* promoter and reactivation of Xp in the extraembryonic lineages. The latter findings indicate that imprinted XCI can be perturbed by altering the control of *Tsix*/*Xist* expression during preimplantation development. In addition, the findings indicate that at least some components of random XCI are shared by the random and imprinted X-inactivation processes.

Recently, on examination of DNMT1o-deficient placentae [26], we detected a higher incidence of and more severe placental defects in female versus male embryos of *Dnmt1^Δ1o/Δ1o^* mothers. These observations suggested that molecular defects extend beyond autosomally imprinted genes and that X chromosome-linked methylation may also be affected following absence of methylation maintenance by DNMT1o at the 8-cell stage. Therefore, we extended our molecular analysis to both the embryo and placenta of male and female *Dnmt1o^mat−/−^* conceptuses, examined methylation at a large number of autosomal and X-linked loci, and traced a DNA hypomethylation defect within the X-inactivation center (*Xic*) back to preimplantation embryos, in blastocysts or shortly after DNMT1o's action in 8-cell embryos. The results uncover a new role for DNMT1o in maintaining methylation on the X chromosome of extraembryonic tissues and in upholding imprinted Xp inactivation.

## Results

### DNMT1o deficiency is associated with sex-specific abnormalities in extraembryonic tissues

**Figure 1:**
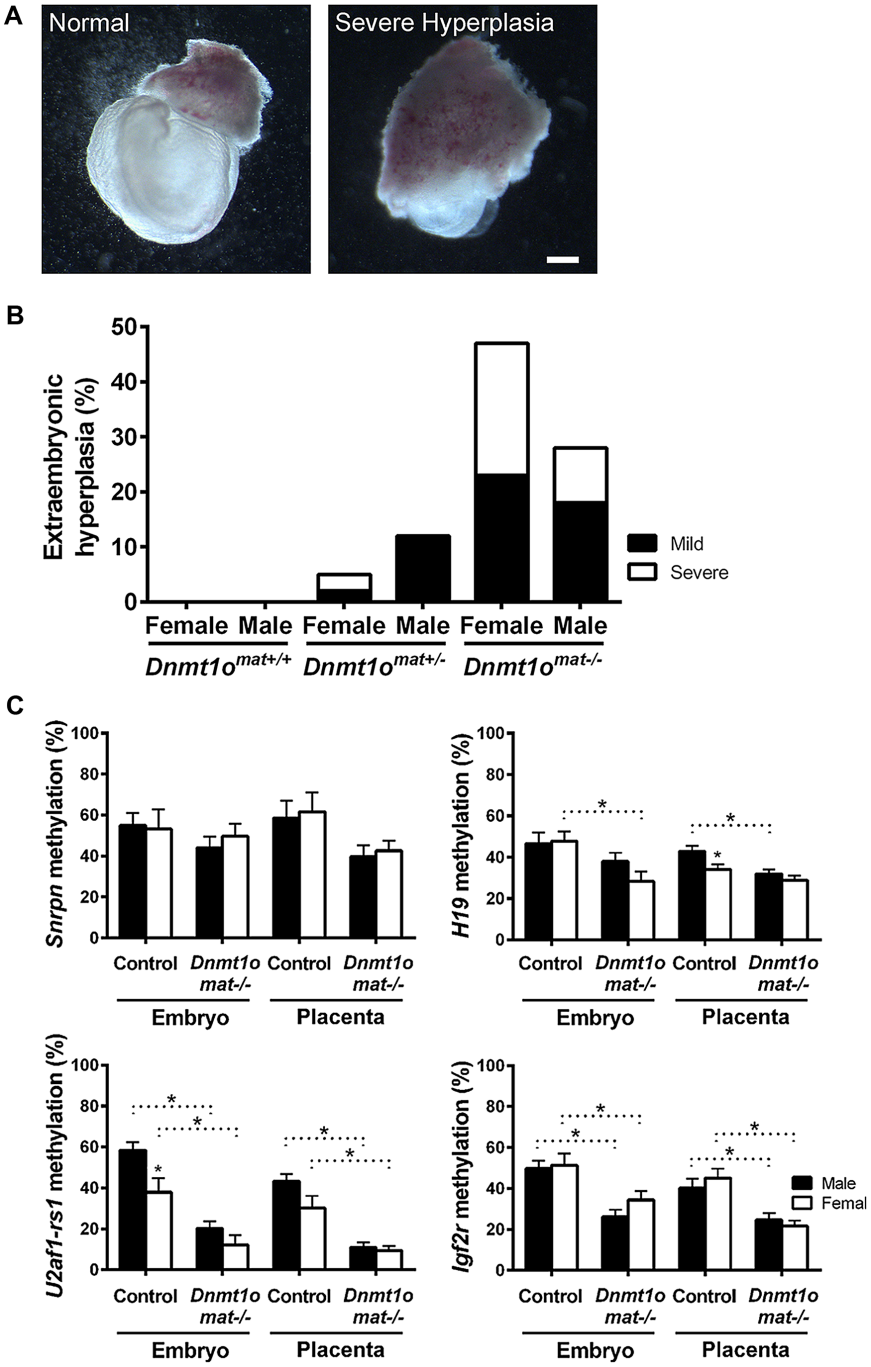
Morphological phenotypes in 9.5dpc extraembryonic tissues associated with DNMT1o deficiency of *Dnmt1o^mat−/−^* offspring. (A) Representative examples of normal and hyperplastic extraembryonic tissues (magnification 16×). Scale bar equals 1 mm. (B) Gross morphological assessment of extraembryonic tissues from *Dnmt1o^mat+/+^* (wild-type), *Dnmt1o^mat+/−^* and *Dnmt1o^mat−/−^* females. Extraembryonic hyperplasia: severe (ectoplacental cone encompassing 2/3 of the embryo), mild (3×8 mm to 6×8 mm), normal (3×4 mm). XX *Dnmt1o^mat−/−^* vs XX *Dnmt1o^mat+/−^*, *p<0.001. (C) Sex-specific analysis of imprinted DMDs in 9.5dpc control and *Dnmt1o^mat−/−^*conceptuses. Analysis of imprinted gene DMD methylation using MassARRAY. Mean ± SEM. p<0.05.

To confirm whether female placentae, versus male placentae, were more severely affected as a result of maternal DNMT1o deficiency, we examined the morphology of extraembryonic tissues of 9.5dpc conceptuses conceived by crossing wild-type males to wild-type *Dnmt1^+/+^*, heterozygous *Dnmt1^Δ1o/+^* and homozygous *Dnmt1^Δ1o/Δ1o^* females. The mutant *Dnmt1^Δ1o^* allele was generated by targeted deletion of exon 1o of the mouse *Dnmt1* gene [12]. Conceptuses from these three crosses are designated *Dnmt1o^mat+/+^*, *Dnmt1o^mat+/−^* and *Dnmt1o^mat−/−^*, respectively. Enlargement or hyperplasia of the extraembryonic tissue layers was a frequent finding, especially amongst female *Dnmt1o^mat−/−^* conceptuses (Figure 1A, Figure S1). *Dnmt1o^mat+/+^* extraembryonic tissues lacked this hyperplasia, whereas *Dnmt1o^mat+/−^* extraembryonic tissues displayed low levels of the abnormality (female: 5%; males: 12%). No case of severe hyperplasia was observed in *Dnmt1o^mat+/−^* males, while severe hyperplasia was found in 3% of *Dnmt1o^mat+/−^* females (Figure 1B). The vast majority of *Dnmt1o^mat+/−^* extraembryonic tissues (92%) were similar in size in both males and females (Table S1). In striking contrast, *Dnmt1o^mat−/−^* extraembryonic tissues displayed a wide range of anatomical abnormalities in both males and females compared to those of *Dnmt1o^mat+/+^* and *Dnmt1o^mat+/−^* embryos (Figure 1B, Table S1, S2, S3). Female *Dnmt1o^mat−/−^* conceptuses showed a major increase in extraembryonic hyperplasia (mild and severe) compared to female *Dnmt1o^mat+/−^* conceptuses (47% vs. 5%, p<0.001). Furthermore, mild and severe hyperplastic abnormalities had a tendency to be more widespread in female extraembryonic tissues compared to their male counterparts (47% vs. 28%, p = 0.1). A detailed histological examination showed that cellular and structural morphology in the *Dnmt1o^mat−/−^* extraembryonic tissue of females was defective at various levels (Figure S2, Supporting Text S1, Table S4, S5). In particular, increases in the giant cell population and abnormal branching to create villi were observed in the extraembryonic tissues of the *Dnmt1o^mat−/−^* females. Thus, a shortage of the maternal DNMT1o enzyme during preimplantation development has more adverse effects on female, as compared to male, placental and extraembryonic tissues.

### Female-specific abnormalities in *Dnmt1o^mat−/−^* extraembryonic tissues are not caused by improper imprinting

Normal placental growth, development and phenotype are largely dependent on proper establishment and maintenance of genomic imprinting (reviewed in [27]). In order to ascertain that the morphological sex bias found in *Dnmt1o^mat−/−^* extraembryonic tissues is independent of imprinting defects, levels of DMD methylation at imprinted loci were quantified. Previously, we provided molecular evidence that oocyte DNMT1o deficiency causes a failure in the maintenance of imprinted DMD methylation in embryos [12], which was more recently extended to include defects in the maintenance of placental DMD methylation [5]. However, sex-specific effects were not investigated in the earlier studies. Here we examined DMD methylation of *H19*, *Snrpn*, *Igf2r* and *U2af1-rs1* in control *Dnmt1o^mat+/+^* and *Dnmt1o^mat−/−^* 9.5dpc embryos and placentae of both males and females (Figure 1C). *Dnmt1o^mat−/−^* animals showed a decrease in placental DMD methylation at all four imprinted loci (p<0.05); however, none of DMDs demonstrated a sex-specific effect in the *Dnmt1o^mat−/−^* group. We conclude from these analyses that autosomal DMD methylation is not affected in a sex-specific manner and that differences in autosomal imprinted gene functions are not responsible for the significant female-specific increase in abnormal placental morphology.

### Abnormal methylation of X-linked genes in female *Dnmt1o^mat−/−^* extraembryonic tissues

**Figure 2:**
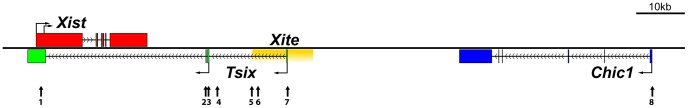
Map of selected elements within or surrounding the X-inactivation center region. The *Xite* (yellow) regulatory element is a positive enhancer of *Tsix* (green) expression, which is an antisense transcript that represses *Xist* (red). Numbers below arrows indicate regions amplified for DNA methylation studies. 1- *Xist*; 2-*Tsix*-CTCFc; 3- *Tsix* CGI, (major promoter); 4- *Tsix* (upstream major promoter); 5- *Xite*-DHS2; 6- *Xite-*DHS4, 7- *Xite*-DHS6 (*Tsix* minor promoter) and 8- *Chic1* promoter.

**Figure 3:**
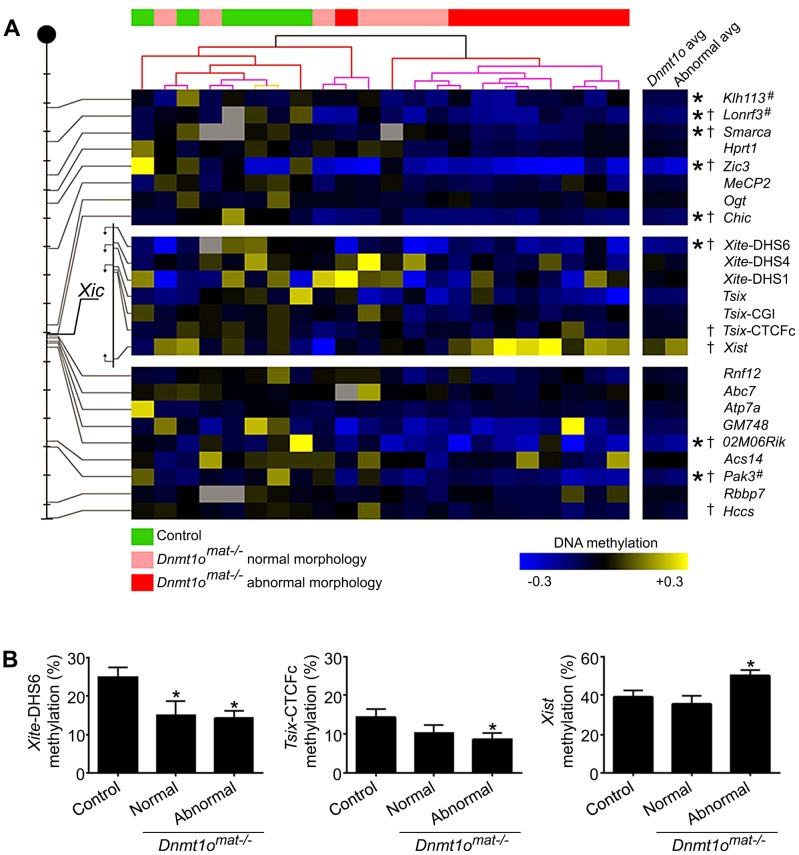
DNA methylation of X-CGI and *Xic* loci in *Dnmt1o^mat−/−^* female placentae. (A) Analysis of X-CGI and *Xic* loci in *Dnmt1o^mat−/−^* female placentae using MassARRAY. The positions of 17 CpG islands and 7 regions of the *Xic* that were analysed are depicted on the left of the figure. Each tick mark equals 10 Mb. Methylation values are displayed relative to the average of the control samples for each gene (blue, hypomethylation; yellow, hypermethylation). Samples are clustered according to their methylation values revealing two distinct groups (green, control; pink, *Dnmt1o^mat−/−^* normal morphology; red, *Dnmt1o^mat−/−^* abnormal morphology). (*p<0.05 control versus *Dnmt1o^mat−/−^*; ^†^p<0.05 control versus *Dnmt1o^mat−/−^* abnormal morphology). Loci marked with “**#**” were identified through RLGS experiments. (B) Histograms displaying the absolute methylation values for *Xite*-DHS6, *Tsix*-CTCFc and *Xist* genes separated into placenta morphology groups (*p<0.05 control versus normal or abnormal *Dnmt1o^mat−/−^*).

Because sex-specific differences in autosomal imprinting could not account for the morphological discrepancy observed between male and female *Dnmt1o^mat−/−^* extraembryonic tissues, we next investigated the possibility that X chromosome DNA methylation is altered. As previously mentioned, the major hallmark of the X chromosome is the unique form of dosage compensation that arises in females to silence one of the two X chromosomes. In the extraembryonic tissues, XCI is imprinted and is thought to be evolutionarily linked to autosomal imprinting [28]–[31]. We carried out an in-depth DNA methylation analysis of multiple CpG dinucleotides at a number of X-linked CpG islands (X-CGIs) distributed throughout the X chromosome and regions of the *Xic* using Sequenom MALDI-TOF mass spectrometry (MassARRAY system). We were particularly interested in CG-rich sequences associated with: 1) *Xite*, an enhancer that positively regulates *Tsix* sustained expression on the active X, 2) *Tsix*, which represses *Xist* expression, and 3) *Xist*, the major effector of the X-inactivation process (Figure 2). *Xite*, *Tsix* and *Xist* are sequences known to display differential methylation between active (Xa) and inactive (Xi) X chromosomes [21]. Data are displayed as the average methylation of all informative CpGs within each amplicon; amplicons were then averaged when more than one was assayed per region (Figure 3A). We compared 17 female 9.5dpc *Dnmt1o^mat−/−^* placentae of various morphologies versus 5 female controls (*Dnmt1o^mat+/+^*). The average methylation for all X-CGIs was significantly decreased in *Dnmt1o^mat−/−^* versus control placentae (p<0.0001) and significant hypomethylation was found in 8/17 individual X-CGIs. X-CGIs that exhibit hypomethylation do not show any positional bias in relation to the *Xic*. For DNA methylation associated with the *Xic*, interestingly, two loci, *Xite*-DHS6 (in *Dnmt1o^mat−/−^* placentae with either normal or abnormal morphology) and *Tsix*-CTCFc (in *Dnmt1o^mat−/−^* placentae with abnormal morphology) revealed significant hypomethylation versus controls (p<0.05) (Figure 3A, B). These two regions are known for their intrinsic enhancer activity on *Tsix* [23], [24]. The 385-bp *Xite*-DHS6 amplicon correlates with important elements for *Tsix* regulation: the *Tsix* 5′ region, which contains the *Tsix* minor promoter and a DNase I hypersensitivity site (DHS), as well as *Tsix* transcriptional start site (TSS) and a substantial part of exon-1 (126 out of 186 bp). The *Tsix*-CTFCc 436-bp amplicon spans exon-3 of *Tsix* and a portion of the DXPas34 repeat, an element crucial in XCI and known to regulate imprinted XCI [32].

Of all regions tested, only *Xist* sequences displayed a trend toward hypermethylation. Because *Dnmt1o^mat−/−^* mice produced conceptuses with morphologically normal and abnormal extraembryonic tissues (Figure 1), we compared the methylation of ten abnormal (mild or severe hyperplasia) *Dnmt1o^mat−/−^* placentae versus controls. In this comparison, along with several X-CGIs that showed significant hypomethylation, *Xist* demonstrated significant hypermethylation (p<0.05) (Figure 3A, B). We next performed a cluster analysis based on the pattern of X-CGI methylation revealed by MassARRAY analysis (Figure 3A). Hierarchal clustering separates the placentae into two primary groups, one containing all 5 of the control placentae and 5 *Dnmt1o^mat−/−^* placentae, in the other all 12 are *Dnmt1o^mat−/−^*. Interestingly, in the group containing the control placentae, only one of the ten samples (a *Dnmt1o^mat−/−^* placenta) is classified as having an abnormal morphology, whereas in the second group 9/12 samples are morphologically abnormal. This distribution is not expected by chance (χ^2^, p<0.01), and suggests a causative relationship between X chromosome hypomethylation and the severity of morphological abnormalities in female *Dnmt1o^mat−/−^* placentae. The DNMT1o-associated X chromosome hypomethylation was restricted to extraembryonic tissue because the same MassARRAY analysis performed on female *Dnmt1o^mat−/−^* embryos versus female control embryos (*Dnmt1o^mat+/+^*) revealed no significant difference in X-linked gene methylation (Figure S3). From these findings, we show that sites across the X chromosome are extensively hypomethylated in female *Dnmt1o^mat−/−^* extraembryonic tissues. The hypomethylation observed in the enhancer regions of *Tsix* and the hypermethylation detected for *Xist* suggest that the imprinted XCI process could be compromised in female *Dnmt1o^mat−/−^* extraembryonic tissues.

### Female-specific preponderance for hypomethylation of single copy loci in *Dnmt1o^mat−/−^* extraembryonic tissues

**Figure 4:**
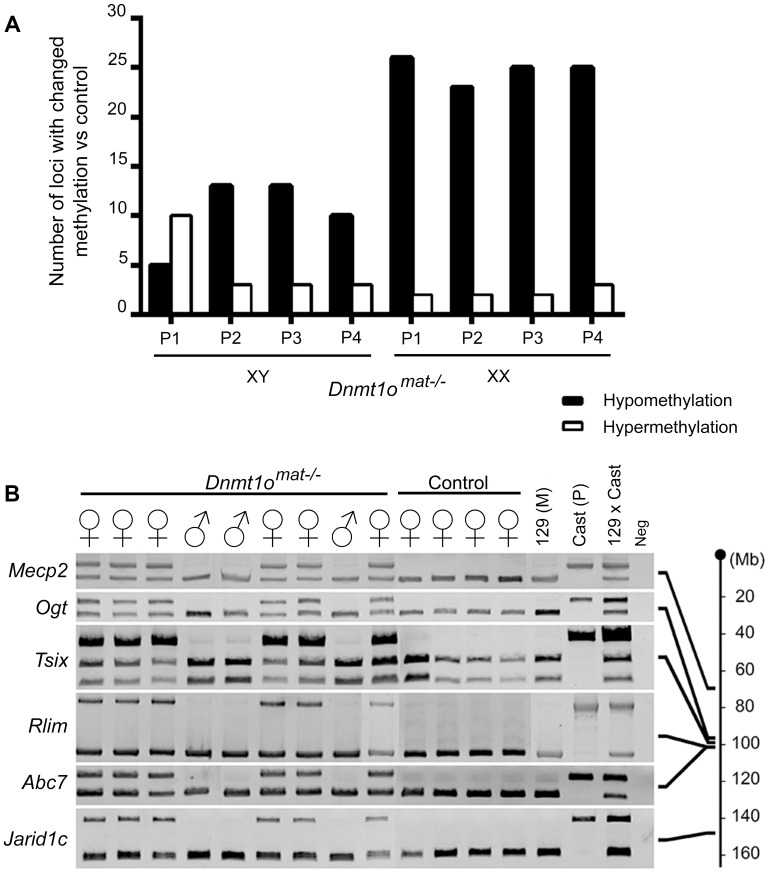
DNA methylation and allelic expression analyses of *Dnmt1o^mat−/−^* female placentae. (A) Sex-specific restriction landmark genomic scanning (RLGS) analysis of single-copy gene DNA methylation in *Dnmt1o^mat−/−^* 9.5dpc placentae. Bars indicate the number of spots showing hypo- and hypermethylation changes in each *Dnmt1o^mat−/−^* placenta (P). (B) Evidence of relaxation of paternal imprinted XCI in female *Dnmt1o^mat−/−^*offspring. Allele specific expression assay (RT-PCR) on X chromosome linked genes. The RNA used was extracted from the visceral endoderm layer of yolk sacs from 9.5dpc *Dnmt1o^mat−/−^* and wild-type control (*Dnmt1o^mat+/+^*) extraembryonic tissues. Maternal (129(M)) and paternal (Cast(P)) control fragments were derived from 129/sv and *Mus musculus castaneus* embryo RNA respectively. The 129× Cast control fragments were generated from F1 hybrid embryo RNA (129/sv × *Mus musculus castaneus*). Schematic on the right shows approximate gene positions (Mb) on the X chromosome.

In parallel, we extended our molecular DNA methylation analysis outside the X chromosome to non-DMD single-copy loci to determine whether DNMT1o deficiency was perturbing other autosomal regions. Restriction Landmark Genomic Scanning (RLGS) 2D-profiles were generated to compare the methylation levels of >2000 single-copy loci between *Dnmt1o^mat+/−^* (control) and *Dnmt1o^mat−/−^* 9.5dpc placentae. This method allows quantification of methylation intensities at genomic NotI restriction sites in CpG islands (CGIs) as well as in non-coding unique and repetitive sequences outside CGIs [33]. Globally, the majority of loci showed a similar methylation level in the placentae of *Dnmt1o^mat−/−^* mice compared to controls, however we uncovered hypo- and hypermethylated sites in both male and female *Dnmt1o^mat−/−^* placentae relative to control placentae (Figure 4A). On average, female *Dnmt1o^mat−/−^* placentae had approximately twice the number of hypomethylated loci (23–26) compared with male *Dnmt1o^mat−/−^* placentae (5–13). Hypermethylation was similar in all *Dnmt1o^mat−/−^* placental samples (2–3 loci), except for placenta XY-P1 where a higher number of hypermethylated loci (10) was observed. Using established RLGS spot cloning methods [34], [35], 18/34 differentially methylated spots in the *Dnmt1o^mat−/−^* placentae were identified (Table 1, Table S6). Loci demonstrating aberrant methylation were distributed among several chromosomes and associated mostly with CGIs and surrounding expressed sequences, either in the 5′region, body or 3′end of genes. A semi-quantitative assessment of changes in spot density shows that methylation varied in the range of 25–50% of normal methylation. Only a single genomic locus, *U2af1-rs1*, was found to be hypomethylated in every female and male sample. *U2af1-rs1* is the only imprinted DMR identified in mouse RLGS profiles using this specific combination of restriction enzymes [36]. Its hypomethylation is consistent with findings on the same gene using the MassARRAY system (Figure 1C). Although none of the hypomethylated loci were identical among the male *Dnmt1o^mat−/−^* placentae, 11 of the 23–26 hypomethylated loci among the female *Dnmt1o^mat−/−^* placentae were identical. We were able to identify 6 of these 11 loci. Intriguingly, all six are positioned on various regions of the X chromosome (Table 1). Spots corresponded to genes *Klhl13*, *Lonrf3*, *Pak3*, *Mum1l1*, *Map3k15* and EST *CJ168414* (or *BY033964*). The hypomethylation observed for *Klhl13*, *Lonrf3* and *Pak3* was validated by MassARRAY over an extended segment (data included in Figure 3A). We conclude from this survey of genomic methylation that although control and *Dnmt1o^mat−/−^* placentae have very similar patterns of genomic methylation, there is concentration of hypomethylated defects on X-linked loci in DNMT1o-deficient placentae.

**Table 1:** Identified loci that display altered DNA methylation in *Dnmt1o^mat−/−^* embryos and placentae.

				Meth. change^a^		Methylation (%)		
Gene (EST)	Chromo. location	CpG island	State (# sample)	XX	XY	Control	*Dnmt1o^mat−/−^*	
*Boll*	chr1: 55,308,150	Y	hypomethylation (4/8)	2	2	50	25	
intergenic	chr10: 37,633,522	N	hypomethylation (6/8)	4	2	90–100	50–75	
*U2af1-rs1*	chr11: 22,924,440	Y	hypomethylation (8/8)	4	4	50	0–10	all^c^
intergenic	chr12: 6,240,843	N	hypomethylation (6/8)	4	2	100	50–75	
intergenic	chr15: 51,913,996	N	hypomethylation (2/8)	1	1	100	75–90	
intergenic	chr19: 44,728,873	Y	hypomethylation (3/8)	3	0	25	0	
*Klhl13*	chrX: 21,798,181	Y	hypomethylation (4/8)	4	0	25	0	all-XX^b^
*Lonrf3*	chrX: 32,760,644	Y	hypomethylation (4/8)	4	0	50–75	25–40	all-XX^b^
*Zic3*	chrX: 53,512,027	Y	hypomethylation (1/8)	1	0	25	0	
*CJ168414*	chrX: 99,368,442	Y	hypomethylation (4/8)	4	0	50	0–25	all-XX^b^
*Mum1l1*	chrX: 134,556,937	N	hypomethylation (4/8)	4	0	50	0	all-XX^b^
*Pak3*	chrX: 138,911,370	N	hypomethylation (4/8)	4	0	50	0	all-XX^b^
*Map3k15*	chrX: 155,332,963	Y	hypomethylation (4/8)	4	0	25	0	all-XX^b^
*Cast1*	chr6: 4,553,959	Y	hypermethylation (2/8)	1	1	0	25–50	
intergenic	chr19: 44,728,873	Y	hypermethylation (1/8)	0	1	25	50	
*Klhl13*	chrX: 21,798,181	Y	hypermethylation (1/8)	0	1	25	50	
*Zic3*	chrX: 53,512,027	Y	hypermethylation (2/8)	0	2	25	50	
*Map3k15*	chrX: 155,332,963	Y	hypermethylation (1/8)	0	1	25	50	

a Number of profiles with methylation change for specific loci (total placenta samples : 4XX + 4XY).

b Methylation changed in all XX profiles and unchanged in all XY profiles.

c Loci methylation changed in all profiles.

### Impairment of paternal imprinted XCI in female *Dnmt1o^mat−/−^* extraembryonic tissues

Because regions within *Xite*, *Xist* and other X-linked genes in *Dnmt1o^mat−/−^* placentae exhibited abnormal methylation, we next determined if paternally imprinted XCI is compromised by the loss of DNMT1o. Firstly, we sought to validate that both the maternal and paternal alleles of X chromosomes in female conceptuses were active in *Dnmt1o^mat−/−^* tissues. To distinguish the parental origin of the active and inactive X chromosome alleles in female *Dnmt1o^mat−/−^* extraembryonic tissues, we generated 9.5dpc conceptuses from inbred 129/Sv mothers (*Dnmt1o^mat−/−^* and control *Dnmt1o^mat+/+^*) and *Mus musculus castaneus* fathers to generate single nucleotide polymorphisms (SNPs) along the X chromosome. This analysis was carried out on visceral endoderm cells where normally the paternal X chromosome is preferentially silenced (imprinted XCI), and gene expression is solely under the control of the active maternal allele [37], [38] (reviewed in [20]). We dissected and isolated the visceral endoderm in order to analyze the expression of six X-linked genes that are situated centromerically (*Mecp2* and *Ogt*) and telomerically (*Rlim*, *Abc7* and *Jarid1c*) to the *Xic* (Figure 4B). As expected, for all six X-linked genes, female *Dnmt1o^mat+/+^* controls exhibited expected expression from only the maternal allele. Male *Dnmt1o^mat−/−^* samples yielded the same banding pattern as they only possess one maternally derived X chromosome. However, female *Dnmt1o^mat−/−^* samples demonstrated biallelic expression for all the X-linked genes (*Mecp2*, *Ogt*, *Tsix*, *Rlim*, *Abc7* and *Jarid1c*), thus showing that the lack of maternal *Dnmt1o* during preimplantation triggers a relaxation event in the imprinted inactivation of the paternal X chromosome.

### Identification of an early hypomethylation event at *Xite* in female *Dnmt1o^mat−/−^* blastocysts

**Figure 5:**
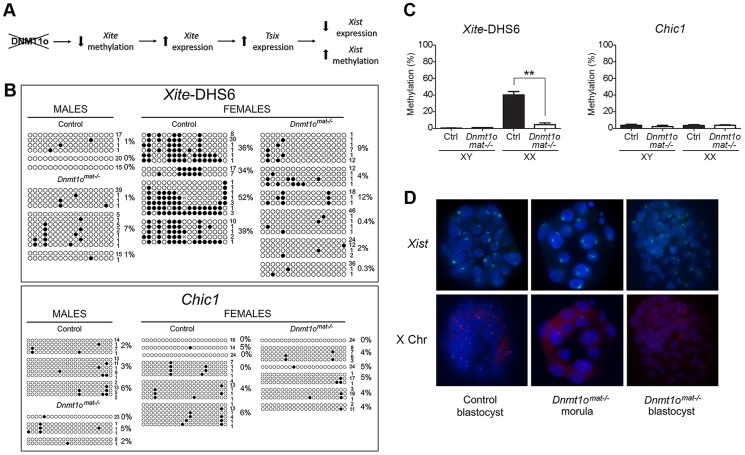
Early *Dnmt1o^mat−/−^* blastocysts exhibit abnormal methylation of *Xite* but normal *Xist* expression. (A) Perturbed imprinted XCI model following *Dnmt1o*-deficiency. Lack of DNMT1o activity initiates hypomethylation events on *Xite* sequences, which activates and sustains the *Tsix* expression on the paternal X chromosome. This chain of events leads to repression of *Xist* expression on the paternal X chromosome. (B) Bisulfite cloning and sequencing results in sexed control and *Dnmt1o^mat−/−^* blastocysts for two regions that exhibited hypomethylation in female *Dnmt1o^mat−/−^* extraembryonic tissues (9.5dpc). Each line represents one sequenced allele, and the number at the left indicates the number of clones sequenced for that allele. Filled circles refer to methylated CpG dinucleotides. Percentages of methylated CpGs are shown. (C) Graphs represent the means of methylation percentages obtained for single sexed blastocysts. Mean ± SEM. **p<0.001. (D) Combined confocal microscopy images from RNA-DNA FISH experiments. Localization of *Xist* RNA (green) marks the inactive X chromosome. Specific X chromosome staining with Dxwas70 (red). Nuclei were counterstained with DAPI (blue).

To identify possible early initiating events that led to the abnormal expression of paternal X-linked genes in female *Dnmt1o^mat−/−^* extraembryonic tissues at 9.5dpc, we examined DNA methylation in preimplantation embryos. Since evidence of Xp genic silencing was reported to occur at the 8- to 16-cell stage [19], in the same time window as DNMT1o action, we postulated that lack of DNMT1o prompted a cascade of events that prevented complete establishment of imprinted XCI in trophectoderm cells of blastocysts (Figure 5A). Given that the interplay among *Xite*, *Tsix* and *Xist* is pivotal in the control of XCI, we investigated if hypomethylation of *Xite* could be the initiating upstream event. In order to reliably sex and perform DNA methylation analysis on X-linked genes from single embryos we examined blastocysts, a stage shortly after the time of action of DNMT1o. A region within *Xite*, *Xite*-DHS6, which exhibited hypomethylation in female *Dnmt1o^mat−/−^* extraembyronic tissues (normal and abnormal morphology, 9.5 dpc) (Figure 3A, B) was selected for classical bisulfite sequencing studies. We postulated that the initial hypomethylation event on the X chromosome would occur within the *Xic*. In the same blastocysts in which we examined *Xite* methylation, we also examined the methylation of *Chic1*, a neighbouring gene that displayed hypomethylation in female *Dnmt1o^mat−/−^* extraembryonic tissues but was located outside the *Xic* (Figure 3A). For *Xite-*DHS6, both control and *Dnmt1o^mat−/−^* male blastocysts revealed very low levels of methylation (Figure 5B, C). In female control blastocysts, the *Xite-*DHS6 loci averaged 40.3% of DNA methylation. In contrast, female DNMT1o-deficient blastocysts had significant DNA hypomethylation (4.6%). For *Chic1*, male and female control (XY-3.6% and XX-3.5%), and *Dnmt1o^mat−/−^* blastocysts (XY-2.3% and XX-3.6%) displayed similar DNA methylation levels (Figure 5B, C).

Next, we used RNA FISH to determine whether the hypomethylation observed for *Xite-*DHS6, a strong *Tsix* enhancer, was associated with altered *Xist* expression in female *Dnmt1o^mat−/−^* blastocysts. First, we carried out *Xist* RNA FISH and subsequently DNA FISH with an X-specific centromeric probe on the same embryos to distinguish male and female embryos. As expected, control female blastocysts showed the characteristic pattern of one *Xist* RNA domain in all cells. Similarly, female *Dnmt1o^mat−/−^* blastocysts also exhibited the same pattern in all cells (Figure 5D). In male control and *Dnmt1o^mat−/−^* blastocysts no *Xist* RNA signals were seen (data not shown). The results provide evidence that although an important regulatory region of *Tsix* is hypomethylated in *Dnmt1o^mat−/−^* embryos, imprinted expression of *Xist* was initiated and persisted until at least the blastocyst stage.

### Hypomethylation of repetitive sequences in *Dnmt1o^mat−/−^* extraembryonic tissues

To determine if additional genomic regions have altered DNA methylation levels in *Dnmt1o^mat−/−^* female placentae, we measured the level of DNA methylation in different retrotransposon sequences. Retrotransposon elements are CpG-rich and characteristically highly methylated, which prevents their transcriptional expression [39]. Roughly half of all mouse genomic sequences are derived from historic transposition events, and the methylation of specific categories of repeated retrotransposon sequences, such as long interspersed nuclear elements (LINE1) and Alu, are often used to gauge the extent of global DNA methylation [40], [41]. Repeat elements are also known to be enriched on X chromosomal DNA compared to the autosomal DNA and are paternally imprinted at the 2-cell stage [19]. One repeat, LINE1, shows a ∼2-fold enrichment (X-linked: 26.5% vs. non-X linked: 13.4%) on the X chromosome, with the uppermost increase measured in the region including the *Xic* [42]. LINE1 has also been implicated in various functions during XCI [19], [42]–[45], although its methylation has not been studied in the context of XCI.

**Figure 6:**
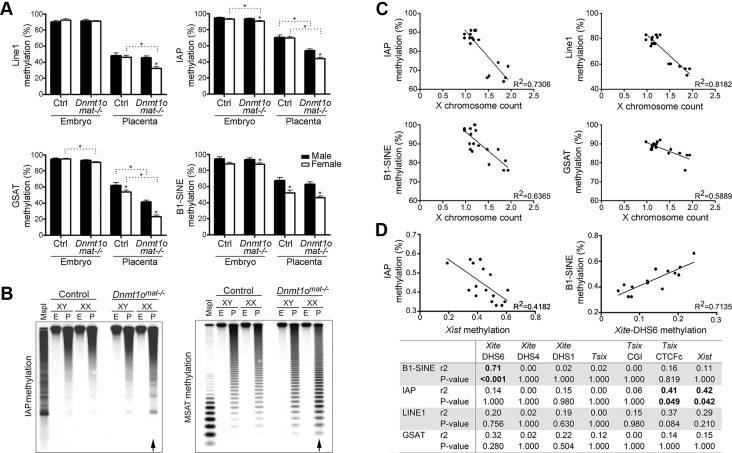
DNA methylation analysis of repeat elements in *Dnmt1o^mat−/−^* 9.5dpc embryos and placentae, as well as in ES cells. (A) Quantification of IAP, GSAT, LINE1, SINE1 repeat sequences methylation using Sequenom MassArray. Partial hypomethylation was observed in control placentae versus embryos for all repeat elements analyzed. Mean ± SEM. *p<0.05. (B) Genomic DNA was digested with MspI (lane I) or HpaII (all other lanes) and hybridized with probes specific to IAP and minor satellite repeats. Arrows identify methylation differences in female placentae between genotypes. (C) Comparison of global repeat methylation and X chromosome count in XX ESC lines. ESC lines were derived from XX *Dnmt1o^mat−/−^* blastocysts. X chromosome complement was measured using real-time PCR of four amplicons located on individual autosomes and four amplicons located on various regions of the X chromosome. Repeat methylation was measured using MassARRAY. (D) Correlation of methylation values of *Xist* with IAP and *Xite*-DHS6 with B1-SINE in *Dnmt1o^mat−/−^* XX-placentae. Repeat and *Xic* methylation were measured in 9.5dpc female placenta samples using MassARRAY. *Xist* methylation was inversely correlated and *Xite*-DHS6 methylation was directly correlated with methylation of repetitive elements. P-values are corrected for multiple comparisons.

Because the *Dnmt1o^mat−/−^* female placentae were hypomethylated at various X-linked loci (Figure 3A, Table 1), we tested whether *Dnmt1o^mat−/−^* female placentae have a reduction in global DNA methylation. We first quantified the methylation status of LINE1, intracisternal A particle (IAP), the major (gamma) satellite repeat (GSAT), the minor satellite repeat (MSAT) and the short-interspersed element (B1-SINE) in placental and embryonic DNA using MassARRAY and Southern blotting (Figure 6A, B, Figure S4). The *Dnmt1o^mat−/−^* genotype did not influence the methylation levels of repeat elements in male or female embryos, which agrees with results from our previous study [12]. However, *Dnmt1o^mat−/−^* female placentae displayed a significant reduction of methylation in 4/5 repeat sequence types versus control placentae. Interestingly, the methylation of all repeats was further reduced in female versus male *Dnmt1o^mat−/−^* placentae. Although repetitive element methylation was not assayed in a chromosome-specific manner using MassARRAY or Southern blotting, the total decrease observed in females versus males is greater than could be accounted for by hypomethylation on the X chromosome alone. Furthermore, RLGS studies revealed in *Dnmt1o^mat−/−^* female placentae, as compared to control females, genomic sites with slight hypomethylation (<25% differences that did not meet the threshold to be counted in Figure 4A and Table 1) that map to various types of interspersed repetitive elements located on various autosomes (Figure S5). These findings reveal a genome-wide repeat methylation defect in *Dnmt1o^mat−/−^* extraembryonic tissue that is accentuated in females.

Analogous to our findings in *Dnmt1o^mat−/−^* extraembryonic tissue, previous work has shown that female (XX) embryonic stem cells (ESCs) derived from mouse blastocysts display global hypomethylation of repetitive sequence elements [46]. This was thought to be the result of the unique metastable condition in which both X chromosomes remained active, as XX ESCs that spontaneously become XO in culture acquired higher methylation levels similar to male (XY) ESCs. In support of this finding, we derived ESC lines from XX wild-type and *Dnmt1o^mat−/−^* blastocysts and found that those ESCs retaining both X chromosomes were hypomethylated at all repeat elements tested (Figure 6C). The combination of ESC and placenta data support a model in which two active X chromosomes are associated with a low level of repeat sequence methylation.

The accentuation of global repeat methylation defect in females derived from oocytes with a maternal shortage of DNMT1o suggests an association to the X chromosome. Intriguingly, we find that repetitive element methylation in *Dnmt1o^mat−/−^* placentae is negatively correlated to *Xist* methylation, which is most strongly demonstrated with IAP repeats (r^2^ = 0.42, p<0.01) (Figure 6D). A positive relationship between *Xite*-DHS6 methylation and B1-SINE methylation (r^2^ = 0.71, p<0.001) was also observed. These findings suggest a positive correlation between the degree of disruption of methylation on the X chromosome as a result of DNMT1o deficiency and the severity of subsequent defects in repeat sequence methylation.

## Discussion

### DNMT1o deficiency generates a unique spectrum of methylation defects

Three main defects in DNA methylation were apparent in *Dnmt1o^mat−/−^* conceptuses derived from *Dnmt1^Δ1o/Δ1o^* mutant oocytes. First, we noted significant reductions in methylation of autosomal DMD sequences in imprinted genes in DNMT1o-deficient conceptuses [5], [12], [14]. Both the embryo and placenta were roughly equally affected. The marked reduction in methylation across all DMD sequences examined is due to a combination of reduced embryonic maintenance methyltransferase activity during preimplantation development and an inability to re-establish methylation *de novo* on DMD sequences in both embryonic and extraembryonic somatic cells. Second, DNMT1o deficiency resulted in a surprising increase in *Xist* methylation in female placentae. The third methylation defect was hypomethylation of genomic sequences. This hypomethylation was found in extraembryonic tissues, most notably on repeated DNA sequences throughout the genome and on single-copy X chromosome sequences of female placentae. Hypomethylation of *Xite* within the *Xic* of female embryos was traced back to preimplantation blastocyst-stage embryos.

Our previous studies have provided us with an excellent understanding for the cause of reduction in DMD methylation in *Dnmt1^mat−/−^* conceptuses. DNMT1o functions at just a single time in early embryonic development, the 8-cell preimplantation stage, and its only known function is maintenance of CpG methylation on genomic sequences. We previously showed that the prominent effect of DNMT1o deficiency was the permanent loss of methylation from one-half of the normally methylated parental alleles of imprinted DMDs. This permanent loss was apparent in both the embryo and placenta. Whether non-imprinted sequences lose methylation in the absence of 8-cell DNMT1o has not been examined. However, if so, the loss in the embryo is not permanent because non-imprinted sequences were methylated to a normal level in mid-gestation *Dnmt1^mat−/−^* embryos ([12] and this report). A return to normal levels of non-imprinted methylation is consistent with the observation that global levels of genomic methylation readily return to normal upon reintroduction of DNMT1 activity in DNMT1s-deficient ES cells [47], [48].

### Source of hyper- and hypomethylated X chromosomal sequences

We can reasonably surmise from the observed biallelic expression of extraembryonic X-linked genes in DNMT1o-deficient conceptuses, including the biallelic expression of *Tsix*, that the paternal X chromosome in 9.5dpc placentae is the source of X-linked hypomethylation and *Xist* hypermethylation. Methylation of *Xist* sequences is a key epigenetic feature that unambiguously distinguishes the active (methylated *Xist*) from the inactive (unmethylated *Xist*) X chromosome in the mouse [49], [50]. For random XCI DNA methylation is not required for the initial repression of *Xist* [51]. Rather, recruitment of DNA methylation to *Xist* sequences is secondary to *Tsix*-mediated recruitment of chromatin modifying enzymes to the *Xist* promoter [17], [52], [53]. It has been shown that *Tsix*-mediated *Xist* repression and promoter methylation operate in extraembryonic cells of the mouse, where XCI is imprinted, or paternal-specific [25], [54]. Based on the strong inverse correlation between *Xist* expression and its methylation, it seems paradoxical that the absence of 8-cell DNMT1o leads to *Xist* methylation on the paternal X chromosome in extraembryonic cells. DNMT1o must therefore act in an indirect way to maintain marks that reinforce imprinted XCI. A possible mode of action is via effects on *Tsix* expression, which is essential for preventing inactivation of the maternal X chromosome in extraembryonic cells with imprinted XCI [25], [54]. The current understanding of XCI is that *Tsix* is controlled by *Xite*, a region that specifically enhances *Tsix* expression [21]–[24]. The *Xite* locus contains three CGIs showing low levels of methylation in oocytes, associated with a single active X, and hypermethylation in sperm, consistent with an inactive paternal X [21]. One of the three CGIs overlaps a DNAse hypersensitive site (*Xite*-DHS6) and the minor *Tsix* promoters. In female ES cells, this region is found undermethylated on both parental alleles, prior to the onset of XCI, however upon cellular differentiation and initiation of XCI, *Xite*-DHS6 becomes hypermethylated on both the maternal and paternal alleles [21]. Considering these and other data, it was suggested that *Xite* might be a candidate for a gametic XCI imprint [21]. Furthermore, *Xite* enhancing action seems influential predominantly at the onset of XCI, since its deletion results in a deficiency of *Tsix* expression at, but not prior to, the onset of random XCI [23].

**Figure 7:**
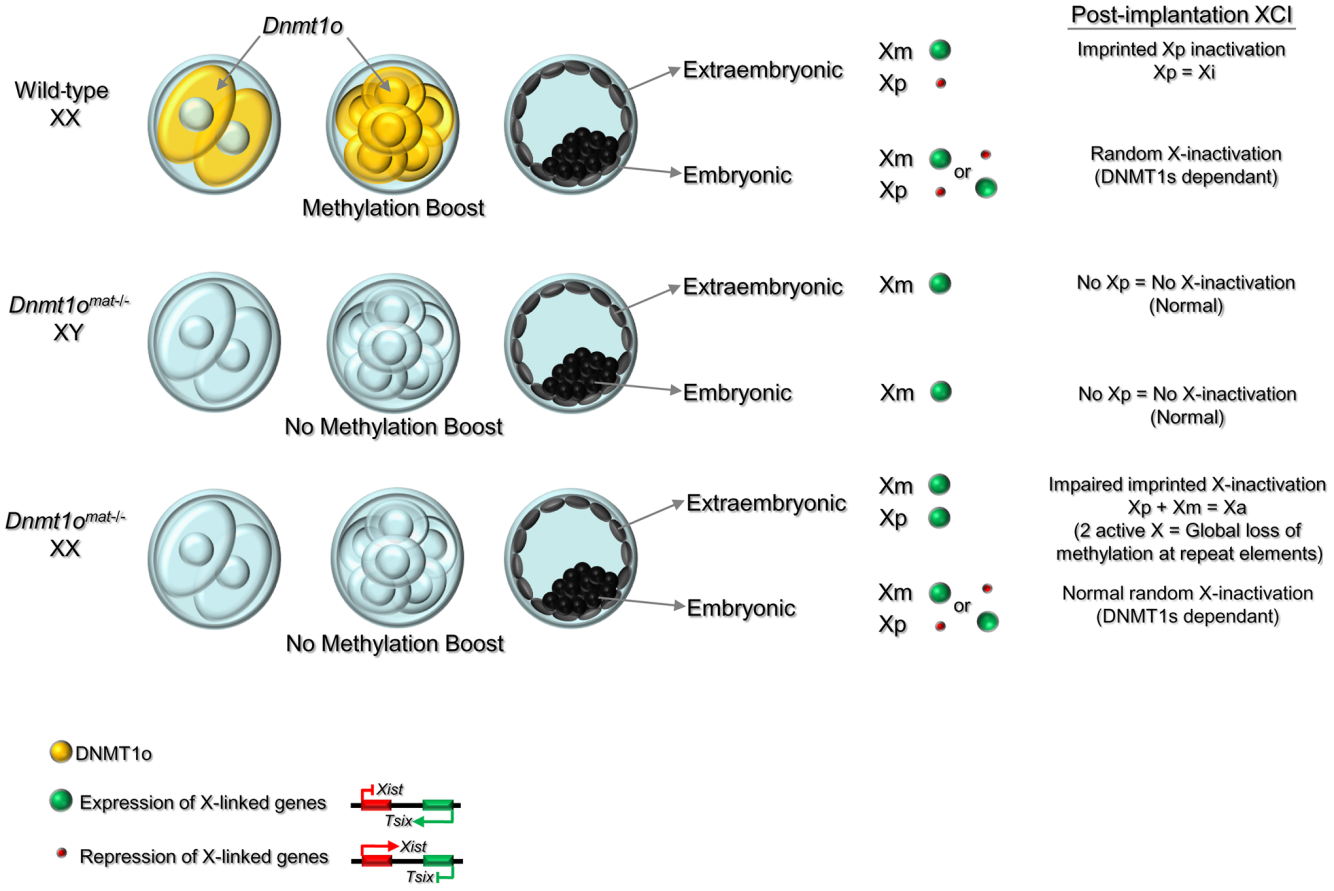
Model of the dynamic regulation of methylation maintenance by DNMT1o and establishment of imprinted XCI during preimplantation development. Wild-type XX: Maternally produced DNMT1o translocates into the nucleus of 8-cell embryos. Nuclear DNMT1o produces a ‘boost’ to maintain methylation marks on DMDs, and sequences on the X chromosome, repeats and other specific sequences. In the blastocyst, a reprogramming and *de novo* methylation phase takes place in the inner cell mass (ICM) [65] and high levels of global genome methylation are observed in this cell lineage. Imprinted XCI is maintained on the Xp in the extraembryonic derivatives, while in the ICM the reprogramming activity reactivates the Xp and random XCI causes inactivation of either the Xm or Xp. *Dnmt1o^mat−/−^* XY and *Dnmt1o^mat−/−^* XX: Lack of DNMT1o at the 8-cell stage prevents the methylation ‘boost’ and causes a failure in the maintenance of methylation marks on DMDs and other sequences including repeat sequences and X-linked genes. This failure of maintenance methylation at the 8-cell stage results in expression from both Xp and Xm in the extraembryonic lineage (relaxation of imprinted XCI). Activation of both X chromosomes in extraembryonic tissues is associated with methylation loss at repeat elements as well as other sequences across the genome. In contrast, the reprogramming event in the ICM restores proper epigenetic patterns and normal random XCI is established in XX embryos. Following the reprogramming and *de novo* methylation phase, the global DNA methylation levels in the XY and XX cells derived from ICM are similar to wild-type.

Based on the aforementioned published models of *Xite* function, we can now understand how DNMT1o and *Xite* converge to regulate imprinted XCI. In our DNMT1o deficiency model, we showed that *Xite*-DHS6 was hypomethylated in XX blastocysts (∼40% vs ∼5%); these results indicate that regions of *Xite* are targeted by DNMT1o for methylation maintenance at the 8-cell stage, the same stage at which Xp genic silencing is initiated. It is expected that loss of *Xite* methylation will be associated with an increase of *Xite* activity, which would lead to *Tsix* expression on the paternal X chromosome; these events would then instigate a process leading to *Xist* repression on the paternal X chromosome (Figure 7 for model). This process does not appear to be immediate, as shown by our RNA FISH results and the persistence of *Xist* RNA expression in *Dnmt1o^mat−/−^* trophectoderm cells of blastocysts. The delay in this proposed *Tsix*-induced *Xist* repression may be due to a developmental program of epigenetic changes thought to occur on *Xist* sequences, culminating in methylation and silencing of its promoter. These last two events need not be coincidental. In this regard, the rapid repression of *Xist* by forced *Tsix* expression on the inactive paternal X chromosome in an inducible transgenic model of *Tsix* expression may be due to supranormal levels of *Tsix* [25]. Nevertheless, even this rapidly induced *Xist* repression is readily reversed, as evidenced by reappearance of *Xist* transcripts when paternal X chromosome *Tsix* expression is turned off. Our results are reminiscent of and may be analogous to those we reported previously for imprinted genes in the *Dnmt1o^mat−/−^* model [5]. While imprinted sequences were hypomethylated in blastocysts, expression remained monoallelic (imprinted) with biallelic expression only seen later in development in 7.5dpc embryos.

A specific effect of DNMT1o methylation on extraembryonic methylation in females might also explain the opposite dependence of DNA methylation on imprinted XCI observed by Sado et al. (2000). They observed that random XCI was abnormal but imprinted XCI was normal in the absence of zygotically produced DNMT1 protein [55]. Our results suggest that such preservation of imprinted XCI in the absence of zygotic DNMT1 could be due to activity of maternally derived stocks of DNMT1 and DNMT1o protein [6] in homozygous *Dnmt1^−/−^* mutant embryos derived from heterozygous *Dnmt1^+/−^* parents.

We must also consider another connection between genome-wide hypomethylation of repeated sequences and loss of extraembryonic imprinted (paternal-specific) XCI in *Dnmt1o^mat−/−^* embryos. It has been proposed that repeated sequences are premarked in male germ cells, which would then be recognized after fertilization to construct a more definitive, permanent paternal-specific mark in extraembryonic cell types [19]. Loss of DNMT1o may directly (if the premark is DNA methylation) or indirectly influence the stability or inheritance of this premark in mid-preimplantation. In one specific model we can propose based on our data, reduction in repeat methylation due to the absence of DNMT1o occurs across the genome (X chromosome and autosomes) and remains reduced upon further development in both male and female extraembryonic cell types, while recovering to normal levels in both male and female embryonic cells. Hypomethylated female XX ES cells, particularly those from DNMT1o-deficient embryos, may model early female *Dnmt1o^mat−/−^* embryonic cells, prior to recovery of genomic methylation and the presumed onset of random XCI. Extraembryonic tissues from *Dnmt1o^mat−/−^* conceptuses and XX ESCs represent two situations where two active X chromosomes are maintained for an unnaturally prolonged period. Both of these conditions result in a global reduction of repeat methylation, and our data also suggests the possibility of a functional relationship between the *Xic* and regulation of global repeat methylation (further discussed in Supporting Text S2).

### Morphological and functional integrity of female and male placentae compromised by DNMT1o deficiency

How can we account for the histomorphological abnormalities in *Dnmt1o^mat−/−^* placentae, including the striking differences between female and male E9.5 *Dnmt1o^mat−/−^* placentae? We can assume that these structural abnormalities are due primarily to methylation abnormalities and their collateral effects, which are most likely transcriptional defects occurring in cells of the developing placentae. Two epigenetic abnormalities were described in *Dnmt1o^mat−/−^* placentae, defects in imprinted DMD methylation, and hypomethylation of repeated sequences and X-linked sequences. DMD methylation abnormalities occurred to the same extent in male and female *Dnmt1o^mat−/−^* E9.5 placentae, and we propose that the histomorphological abnormalities in male *Dnmt1o^mat−/−^* placentae were due entirely or nearly entirely (because there was hypomethylation of repeated sequence, presumably autosomal, in male *Dnmt1o^mat−/−^* placentae) to such DMD methylation abnormalities. It is known that disruptions in the integrity of the strict parent-specific methylation of imprinted DMD sequences can lead to dysregulation of imprinted gene expression, including dysregulation of placentally expressed imprinted genes. Disruption in the monoallelic imprinted expression of individual placentally expressed genes can result in profound histomorphological abnormalities [20] and therefore it is likely that dysregulation of imprinted gene expression alone in *Dnmt1o^mat−/−^* placentae could account for all of the male *Dnmt1o^mat−/−^* morphological defects.

We further propose that hypomethylation of X chromosome sequences in female *Dnmt1o^mat−/−^* placentae accounts for the histomorphological abnormalites unique to the female *Dnmt1o^mat−/−^* placentae. How might this X chromosome hypomethylation lead to gross and microscopic morphological abnormalities? A likely scenario is that this hypomethylation initially occurred at the time of, or soon after, the loss of DNMT1o in preimplantation development, and that it persisted in extraembryonic cell types (see above). The hypomethylation, via an unknown mechanism, would lead to dysregulation in the expression of X-linked genes and associated female-specific extraembryonic morphological abnormalities. Failure in imprinted XCI via the deregulation of *Xist* is known to lead to morphological aberrations. Female conceptuses that contain an *Xist* deletion by paternal transmission have severe growth retardation and die in early embryogenesis [56]. Some *Xist^−/−^* conceptuses that fail to undergo gastrulation show elongated ectoplacental cones and expanded yolk sacs. Authors stipulate that the instability caused by the two active X chromosomes is responsible for the imprinted lethal phenotype observed in the mutant *Xist* females.

Since in the *Dnmt1o^mat−/−^* females, only 50% of the placental cells are affected, one might ask why loss of imprinted XCI in 50% of the cells is not compensated by the cells presumably retaining imprinted XCI. Evidence from a recent study suggests that compensation does occur. Thus while there were clear female-specific placental abnormalities at 9.5dpc (this study), by 17.5 dpc there were no differences in the embryo-to-placenta weight ratios or placental phenotypes between *Dnmt1o^mat−/−^* males and females [26]. The exact nature of this compensation is unknown, but we can speculate that it is a combination of viability of 50% of cells retaining normal imprinted X-inactivation, death of many cells not expressing *Xist* and compensatory random X-inactivation that rescues a fraction of the *Xist* non-expressors.

In conclusion, the work presented here furthers our understanding on the maintenance of methylation patterns during early embryo development. This study is the first to provide evidence of additional roles for DNMT1o beyond the maintenance of genomic imprinting in preimplantation embryos, including an unexpected role in imprinted XCI. Although further investigation is needed to comprehend how DNA methylation regulates *Xite* and the mechanisms behind the relaxation of imprinted XCI, these findings provide novel mechanistic concepts into the counting mechanism.

## Materials and Methods

Details of experimental procedures are provided in the Supporting Information section (Supporting Text S3).

### Ethics statement

All animal procedures were carried out in accordance with the Canadian Council of Animal Care.

### Assessment of extraembryonic hyperplasia and histological analysis

Embryos and placentae of different genotypes (*Dnmt1o^mat+/+^*, *Dnmt1o^mat+/−^*, *Dnmt1o^mat−/−^*) were collected from the uteri at 9.5dpc and extraembryonic hyperplasia was assessed by microscopy. For the histological analysis, fixed 9.5dpc implantation sites were sectioned and stained with hematoxylin/eosin (H&E).

### DNA methylation analyses

Quantitative DNA methylation analyses were accomplished using isolated and bisulfite treated DNA from 9.5dpc *Dnmt1o^mat+/+^* and *Dnmt1o^mat−/−^* embryos and placentae. Candidate regions of imprinted DMDs, X chromosome-CGI and repeat elements were amplified (primers see Table S7) and the quantitative DNA methylation state was analyzed using matrix-assisted laser desorption ionization time-of-flight (MALDI-TOF) mass spectrometry [57].

Sanger bisulfite sequencing was used to analyzed the methylation status in single male and female control (*Dnmt1o^mat+/+^*) and mutant (*Dnmt1o^mat−/−^*) 3.5dpc blastocysts. Isolated DNA was modified by sodium bisulfite, and *Xite-*DHS6 and *Chic1* were amplified by PCR (primers see Table S7), subcloned and sequenced.

Restriction landmark genomic scanning (RLGS) was used to assess genome-wide methylation in 9.5dpc control (*Dnmt1o^mat+/−^*) and *Dnmt1o^mat−/−^* placentae [33]. Sufficient DNA was obtained from single control and *Dnmt1o^mat−/−^* 9.5dpc placentae, however in the case of smaller samples, pools of similar placentae (same sex and similar morphology) were used to obtain sufficient tissue for DNA extraction and RLGS studies (samples XY P4 and XX P4).

Southern blots on 9.5dpc *Dnmt1o^mat+/+^* and *Dnmt1o^mat−/−^* placentae were performed as described [58] and visualized by autoradiography. Minor satellite probes were constructed by PCR amplification of mouse genomic DNA [59] as previously described [60], [61]. DNA was digested with either MspI or its methylation-sensitive isoschizomer HpaII. Membranes were stripped and reprobed according to the manufacturer's recommended conditions (Hybond; GE Healthcare/Amersham).

### RNA and DNA FISH

RNA and DNA FISH were performed, as previously described [62], on *Dnmt1o^mat+/+^* and *Dnmt1o^mat−/−^* preimplantation embryos using an *Xist* probe (pEFB, [63]) and a X chromosome probe (DXwas70, [64]).

### Allele specific expression assay by RT-PCR

RNA was extracted from the visceral endoderm layers of *Dnmt1o^mat+/+^* and *Dnmt1o^mat−/−^* yolk sacs from 9.5dpc conceptuses. Following reverse transcription, X chromosome linked genes containing polymorphisms were amplified with the use of flanking primers (Table S7), and the PCR products were digested and separated on acrylamide gels.

### Statistical analysis

Comparisons in the extraembryonic hyperplasia and histological studies were first subjected to Levene's test for homogeneity of variance. One-way ANOVA was used for equal variance groups, while the non-parametric test Kruskal-Wallis was applied to unequal variance groups. Comparisons in methylation of imprinted DMDs and repeat elements were made using the Holm-Sidak method for multiple pair-wise comparisons, whereas analysis of X-CGI methylation was accomplished using an unprotected t test of the difference between means.

## Supporting Information

Figure S1 (Related to Figure 1A, B). Morphological phenotypes in 9.5dpc extraembryonic tissues associated with DNMT1o deficiency of *Dnmt1o^mat−/−^* offspring. Representative examples of normal, mild and hyperplastic extraembryonic tissues (magnification 16×). Scale bar equals 1 mm.
(TIF)

Figure S2 (Related to Figure 1). Extraembryonic histological defects associated with DNMT1o deficiency in *Dnmt1o^mat−/−^* offspring. (A) Shown are Hematoxylin/Eosin (H&E) stained sections of 9.5 dpc implantation sites: representative sections of the various phenotypes observed in the offspring of control (wild-type) and *Dnmt1o^mat−/−^* females. Panel: i) male, ii) male, iii) female and iv) female. al: allantois, ch: chorion, de: decidua, gi: giant cell layer, sp: spongiotrophoblast cell layer. (B) The total number, in the 9.5dpc H&E sections, of trophoblast giant cells (TGCs) per section and the extent of branching in the labyrinth (0 = absent, 1 = initiated, 2 = intermediate, 3 = normal (same as controls)). Mean + SEM. Different letters indicate p<0.05. Scale bar 10 µm.
(TIF)

Figure S3 (Related to Figure 3). Methylation quantification of X chromosome linked genes in embryos. Sequenom MassArray methylation profiles along the X chromosome in female 9.5dpc wild-type (control) and *Dnmt1o^mat−/−^* embryos. Last number following gene name indicates primer set. Illustrated on the right: map of X-linked genes analysed.
(TIF)

Figure S4 (Related to Figure 6B). DNA methylation quantification of IAP and minor satellite repeat sequences in *Dnmt1o^mat−/−^* embryos and placentae. Densitometry measurements from Southern blot gels in Figure 6B.
(TIF)

Figure S5 (Related to Table 1). Analysis of faintly hypomethylated spots in female *Dnmt1o^mat−/−^* 9.5dpc placentae using RLGS. (A) A large number of faint spots (<25% hypomethylation) are present in control placenta RLGS profiles. In *Dnmt1o^mat−/−^* placenta profiles there are many spots that increase in intensity and are marked by black arrowheads a–q (note: faint spot changes were excluded from the primary RLGS analysis). (B) Identification of approximately half of the spots displayed here reveals hypomethylation of various types of interspersed repeats on various chromosomes.
(TIF)

Table S1 (related to Figure 1B). Hyperplasia assessment of 9.5dpc extraembryonic tissues from litters of *Dnmt1o^mat+/−^* females.
(DOCX)

Table S2 (related to Figure 1B). Hyperplasia assessment of 9.5dpc extraembryonic tissues from litters of *Dnmt1o^mat+/+^* females.
(DOCX)

Table S3 (related to Figure 1B). Hyperplasia assessment of 9.5dpc extraembryonic tissues from litters of *Dnmt1o^mat−/−^* females.
(DOCX)

Table S4 (related to Figure S2B). Extent of branching patterns in labyrinth of 9.5dpc extraembryonic tissues from litters of control and *Dnmt1o^mat−/−^* females.
(DOCX)

Table S5 (related to Figure S2B). Trophoblast giant cells per section in 9.5dpc extraembryonic tissues from litters of control and *Dnmt1o^mat−/−^* females.
(DOCX)

Table S6 (related to Table 1). Unidentified loci that display altered DNA methylation in *Dnmt1o^mat−/−^* embryos and placentas.
(DOCX)

Table S7 List of primer sets and sequences used in PCR based experiments.
(DOCX)

Text S1 Supporting Results details. DNMT1o deficiency is associated with sex-specific abnormalities in extraembryonic tissues.
(DOCX)

Text S2 Supporting Discussion details. Relationship between global B1-SINE methylation and the *Xic*.
(DOCX)

Text S3 Supporting Materials and Methods.
(DOCX)
