# Supplementary figures and images for: Loss of DNMT1o Disrupts Imprinted X Chromosome Inactivation and Accentuates Placental Defects in Females
Source: PLoS Genet. 2013 Nov 21;9(11):e1003873. doi: 10.1371/journal.pgen.1003873 (PMC3836718; doi:10.1371/journal.pgen.1003873)

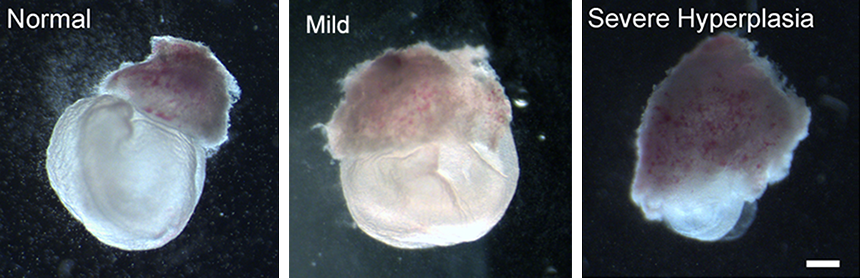

Supplement: Figure S1 — (Related to Figure 1A, B). Morphological phenotypes in 9.5dpc extraembryonic tissues associated with DNMT1o deficiency of Dnmt1omat−/− offspring. Representative examples of normal, mild and hyperplastic extraembryonic tissues (magnification 16×). Scale bar equals 1 mm. (TIF) [file pgen.1003873.s001.tif]

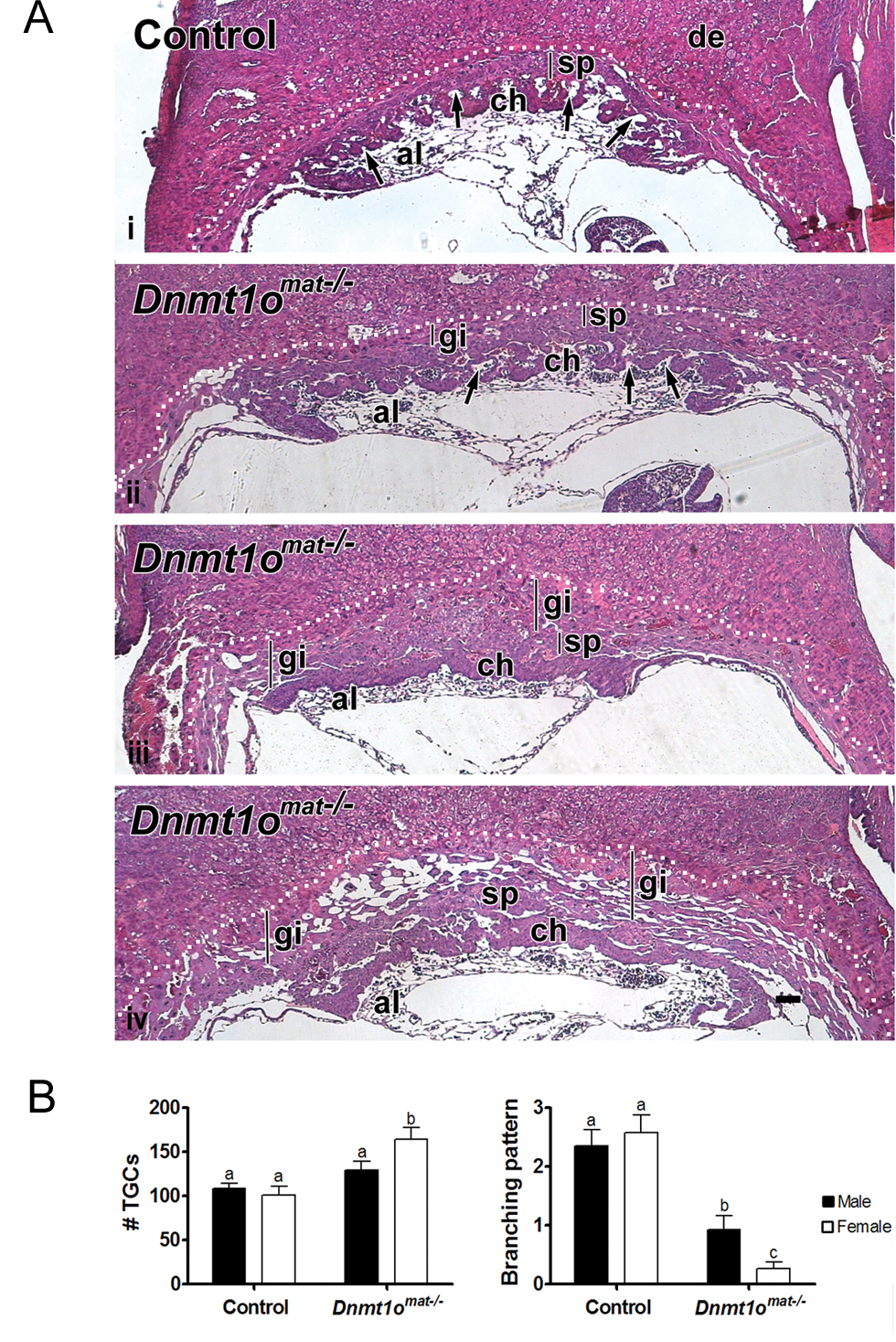

Supplement: Figure S2 — (Related to Figure 1). Extraembryonic histological defects associated with DNMT1o deficiency in Dnmt1omat−/− offspring. (A) Shown are Hematoxylin/Eosin (H&E) stained sections of 9.5 dpc implantation sites: representative sections of the various phenotypes observed in the offspring of control (wild-type) and Dnmt1omat−/− females. Panel: i) male, ii) male, iii) female and iv) female. al: allantois, ch: chorion, de: decidua, gi: giant cell layer, sp: spongiotrophoblast cell layer. (B) The total number, in the 9.5dpc H&E sections, of trophoblast giant cells (TGCs) per section and the extent of branching in the labyrinth (0 = absent, 1 = initiated, 2 = intermediate, 3 = normal (same as controls)). Mean + SEM. Different letters indicate p<0.05. Scale bar 10 µm. (TIF) [file pgen.1003873.s002.tif]

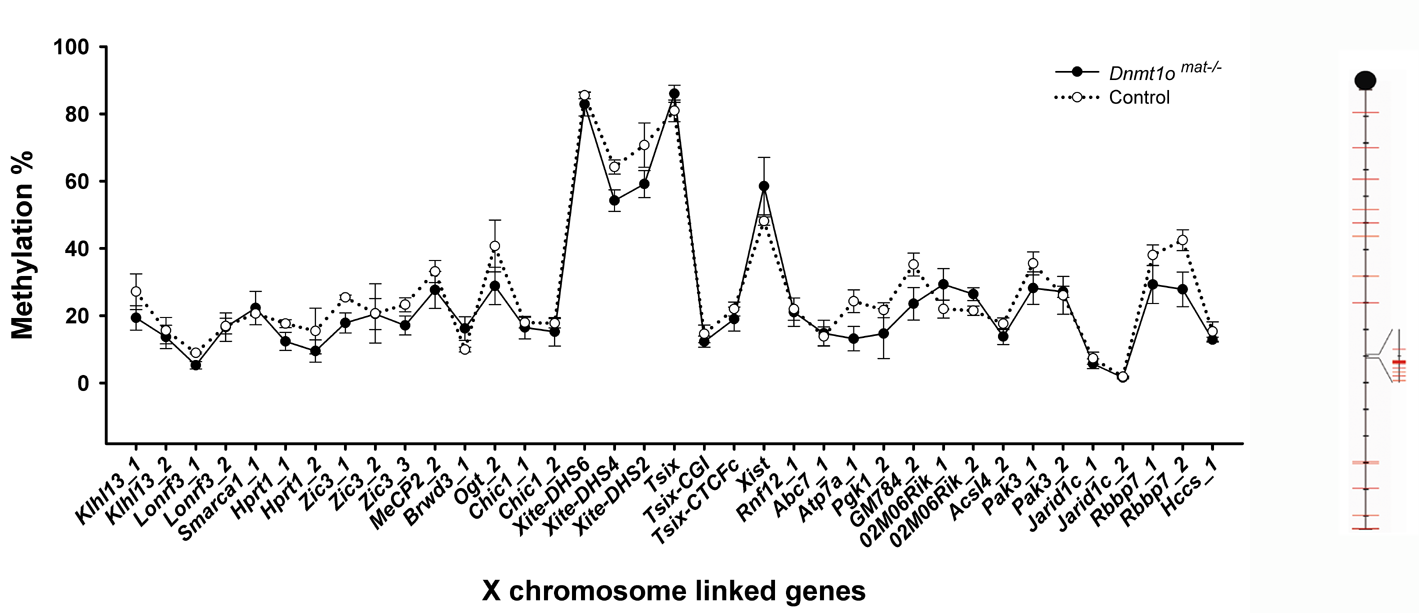

Supplement: Figure S3 — (Related to Figure 3). Methylation quantification of X chromosome linked genes in embryos. Sequenom MassArray methylation profiles along the X chromosome in female 9.5dpc wild-type (control) and Dnmt1omat−/− embryos. Last number following gene name indicates primer set. Illustrated on the right: map of X-linked genes analysed. (TIF) [file pgen.1003873.s003.tif]

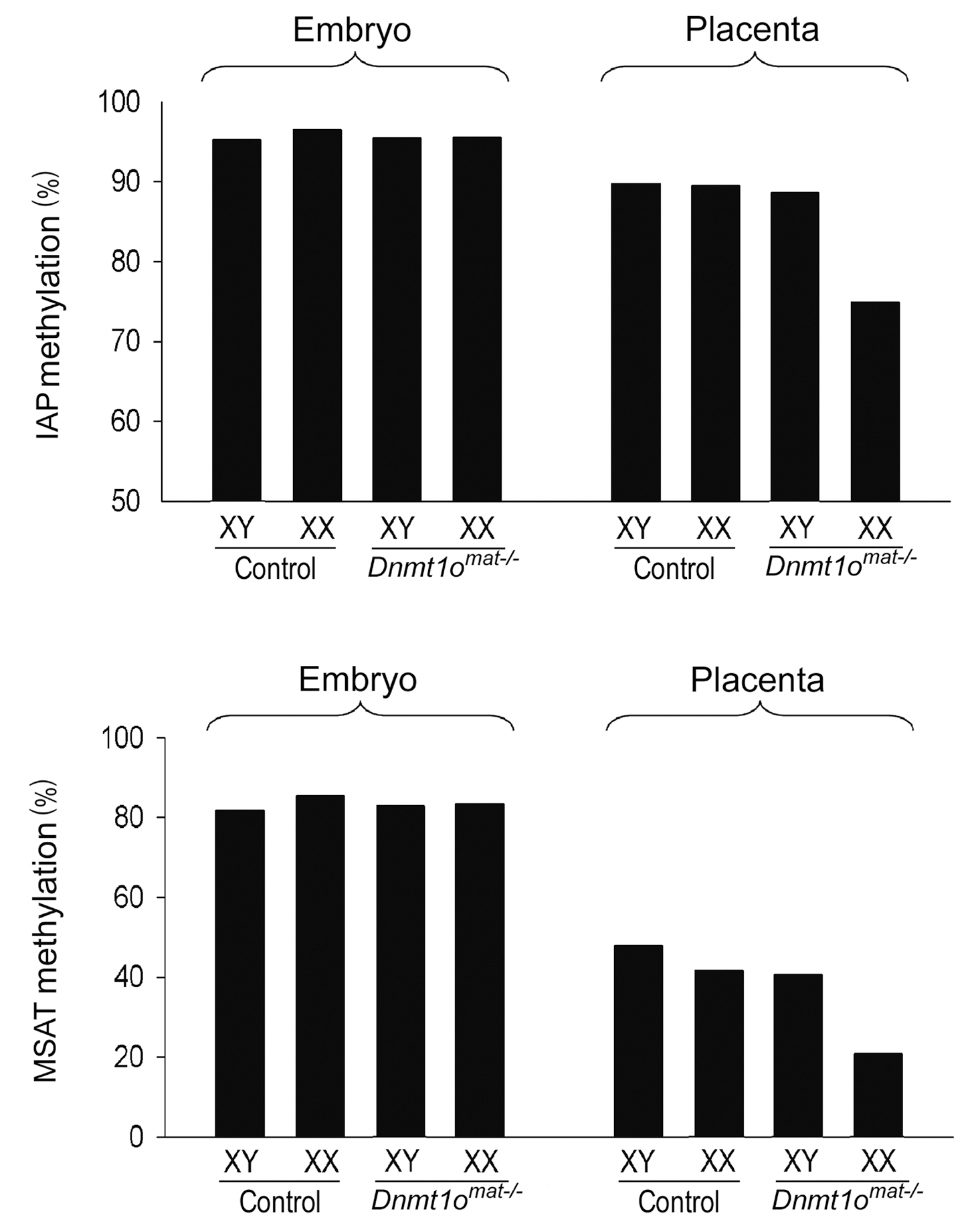

Supplement: Figure S4 — (Related to Figure 6B). DNA methylation quantification of IAP and minor satellite repeat sequences in Dnmt1omat−/− embryos and placentae. Densitometry measurements from Southern blot gels in Figure 6B. (TIF) [file pgen.1003873.s004.tif]

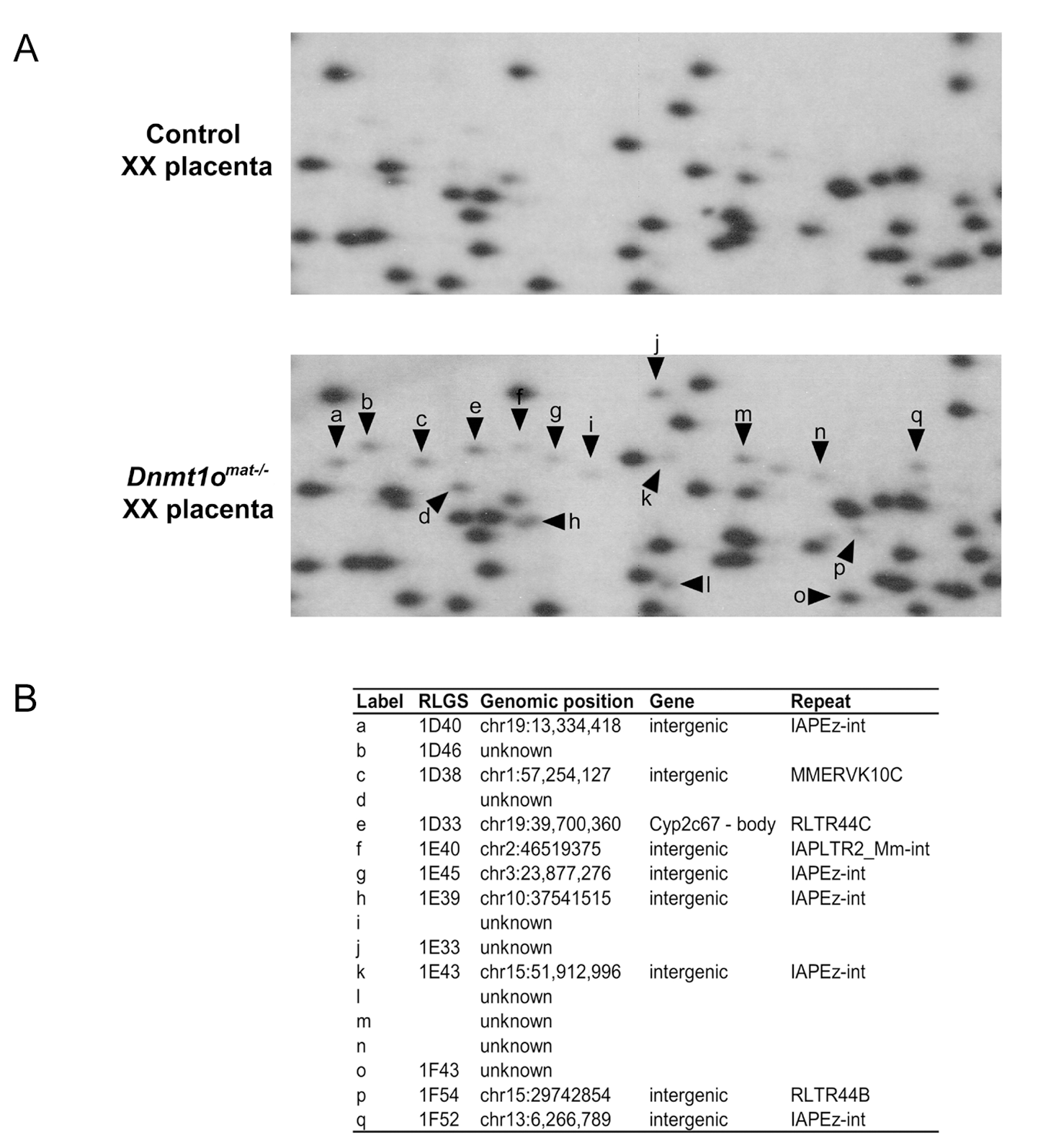

Supplement: Figure S5 — (Related to Table 1). Analysis of faintly hypomethylated spots in female Dnmt1omat−/− 9.5dpc placentae using RLGS. (A) A large number of faint spots (<25% hypomethylation) are present in control placenta RLGS profiles. In Dnmt1omat−/− placenta profiles there are many spots that increase in intensity and are marked by black arrowheads a–q (note: faint spot changes were excluded from the primary RLGS analysis). (B) Identification of approximately half of the spots displayed here reveals hypomethylation of various types of interspersed repeats on various chromosomes. (TIF) [file pgen.1003873.s005.tif]
