# Supplementary material for: Loss of DNMT1o Disrupts Imprinted X Chromosome Inactivation and Accentuates Placental Defects in Females
Source: PLoS Genet. 2013 Nov 21;9(11):e1003873. doi: 10.1371/journal.pgen.1003873 (PMC3836718; doi:10.1371/journal.pgen.1003873)
Supplement: Table S1 — (related to Figure 1B). Hyperplasia assessment of 9.5dpc extraembryonic tissues from litters of Dnmt1omat+/− females. (DOCX) [file pgen.1003873.s006.docx]

| **Table S1 (related to Figure 1B).** Hyperplasia assessment of 9.5dpc extraembryonic tissues from litters of *Dnmt1o^mat+/-^* females.  **A**  **A** | | | | |
| --- | --- | --- | --- | --- |
|  |  | Proportion of Extraembryonic  Hyperplasia | | Degree of |
| Litter # | # Embryos | Females | Males | Hyperplasia * |
| ***Dnmt1o^mat+/-^*** |  |  |  |  |
| 1 | 8 | 0 / 5 | 1 / 3 | Mild |
| 2 | 7 | 0 / 3 | 0 / 4 | --- |
| 3 | 7 | 0 / 3 | 2 / 4 | Mild |
| 4 | 8 | 1 / 4 | 0 / 4 | Mild |
| 5 | 5 | 0 / 5 | --- | --- |
| 6 | 8 | 0 / 3 | 1 / 5 | Mild |
| 7 | 10 | 1 / 4 | 0 / 6 | Severe |
| 8 | 7 | 0 / 5 | 0 / 2 | --- |
| 9 | 9 | 0 / 6 | 0 / 3 | --- |
| 10 | 4 | 0 / 1 | 0 / 3 | --- |
| Rate of all Hyperplasia: | | 2 / 39 (5%) | 4 / 34 (12%) |  |
| Rate of Severe Hyperplasia: | | 1 / 39 (3%) | 0 / 34 (0%) |  |
| Total Hyperplasia XX+XY: | | 6 / 73 (8%) |  |  |

* Mild Hyperplasia: 3x8 mm to 6x8 mm (control = 3x4 mm)

Severe Hyperplasia: Ectoplacental Cone Encompassing 2/3 of the Embryo.
