# Supplementary material for: Loss of DNMT1o Disrupts Imprinted X Chromosome Inactivation and Accentuates Placental Defects in Females
Source: PLoS Genet. 2013 Nov 21;9(11):e1003873. doi: 10.1371/journal.pgen.1003873 (PMC3836718; doi:10.1371/journal.pgen.1003873)
Supplement: Table S2 — (related to Figure 1B). Hyperplasia assessment of 9.5dpc extraembryonic tissues from litters of Dnmt1omat+/+ females. (DOCX) [file pgen.1003873.s007.docx]

| **Supplemental Table S2 (related to Figure 1B).** Hyperplasia assessment of 9.5dpc extraembryonic tissues from litters of *Dnmt1o^mat+/+^* females.  **A**  **A** | | | | |
| --- | --- | --- | --- | --- |
|  |  | Proportion of Extraembryonic  Hyperplasia | | Degree of |
| Litter # | # Embryos | Females | Males | Hyperplasia * |
| ***Dnmt1o^mat+/+^*** |  |  |  |  |
| 1 | 9 | 0 / 2 | 0 / 7 | --- |
| 2 | 6 | 0 / 3 | 0 / 3 | --- |
| 3 | 9 | 0 / 6 | 0 / 3 | --- |
| 4  5  6 | 7  10  7 | 0 / 3  0 / 4  0 / 4 | 0 / 4  0 / 6  0 / 3 | ---  ---  --- |
| Rate of all Hyperplasia: | | 0 / 22 (0%) | 0 / 26 (0%) |  |
| Rate of Severe Hyperplasia: | | 0 / 22 (0%) | 0 / 26 (0%) |  |
| Total Hyperplasia XX+XY: | | 0 / 48 (0%) |  |  |

* Mild Hyperplasia: 3x8 mm to 6x8 mm (control = 3x4 mm)

Severe Hyperplasia: Ectoplacental Cone Encompassing 2/3 of the Embryo.
