# Supplementary material for: Loss of DNMT1o Disrupts Imprinted X Chromosome Inactivation and Accentuates Placental Defects in Females
Source: PLoS Genet. 2013 Nov 21;9(11):e1003873. doi: 10.1371/journal.pgen.1003873 (PMC3836718; doi:10.1371/journal.pgen.1003873)
Supplement: Table S3 — (related to Figure 1B). Hyperplasia assessment of 9.5dpc extraembryonic tissues from litters of Dnmt1omat−/− females. (DOCX) [file pgen.1003873.s008.docx]

| **Table S3 (related to Figure 1B)**. Hyperplasia assessment of 9.5dpc extraembryonic tissues from litters of *Dnmt1o^mat-/-^* females. | | | | |
| --- | --- | --- | --- | --- |
|  |  | Proportion of Extraembryonic Hyperplasia | | Degree of |
| Litter # | # Embryos | Females | Males | Hyperplasia * |
| 1 | 5 | 1 / 3 | 0 / 2 | Severe |
| 2 | 5 | 1 / 4 | 0 / 1 | Severe |
| 3 | 4 | 2 / 3 | 0 / 1 | Mild |
|  |  | 1 / 3 | 0 / 1 | Severe |
| 4 | 5 | 0 / 2 | 1 / 3 | Mild |
| 5 | 4 | 1 / 2 | 0 / 2 | Severe |
| 6 | 3 | 0 / 0 | 0 / 3 | --- |
| 7 | 3 | 1 / 1 | 1 / 2 | Mild |
| 8 | 8 | 0 / 4 | 1 / 4 | Mild |
|  |  | 2 / 4 | 3 / 4 | Severe |
| 9 | 1 | --- | 0 / 1 | --- |
| 10 | 1 | --- | 1 / 1 | Mild |
| 11 | 1 | --- | 0 / 1 | --- |
| 12 | 2 | 1 / 2 | --- | Mild |
|  |  | 1 / 2 | --- | Severe |
| 13 | 5 | 1 / 4 | 0 / 1 | Mild |
| 14 | 6 | 0 / 1 | 1 / 5 | Mild |
| 15 | 7 | 1 / 3 | 1 / 4 | Severe |
| 16 | 8 | 2 / 4 | 1 / 4 | Mild |
| 17 | 8 | 2 / 5 | 1 / 4 | Mild |
|  |  | 1 / 5 | 0 / 4 | Severe |
| Rate of all Hyperplasia: | | 18 / 38 (47%) | 11 / 39 (28 %) |  |
| Rate of severe Hyperplasia: | | 9 / 38 (24%) | 4 / 39 (10 %) |  |
| Total Hyperplasia XX+XY: | | 29 / 77 (38%)** |  |  |

* Mild Hyperplasia: 3x8 mm to 6x8 mm (control = 3x4 mm)

Severe Hyperplasia: Ectoplacental Cone Encompassing 2/3 of the Embryo

** p<0.001, *Dnmt1o^mat-/-^ vs Dnmt1o^mat+/+^* and *Dnmt1o^mat-/-^ vs Dnmt1o^mat+/-^*

Conceptuses from *Dnmt1o^mat+/+^* and *Dnmt1o^mat+/-^* used for statistical analysis are from Table S1 and S2.
