# Supplementary material for: Loss of DNMT1o Disrupts Imprinted X Chromosome Inactivation and Accentuates Placental Defects in Females
Source: PLoS Genet. 2013 Nov 21;9(11):e1003873. doi: 10.1371/journal.pgen.1003873 (PMC3836718; doi:10.1371/journal.pgen.1003873)
Supplement: Table S4 — (related to Figure S2B). Extent of branching patterns in labyrinth of 9.5dpc extraembryonic tissues from litters of control and Dnmt1omat−/− females. (DOCX) [file pgen.1003873.s009.docx]

| **Table S4 (related to Figure S2B)**. Extent of branching patterns in labyrinth of 9.5dpc extraembryonic tissues from litters of control and *Dnmt1o^mat-/-^* females. | | | | | | | |
| --- | --- | --- | --- | --- | --- | --- | --- |
|  |  | |  | |  | |  |
|  | Branching Pattern* | | | | | | |
|  | Control | | | *Dnmt1o^mat-/-^* | | | |
| Placenta | Male | Female | | Male | | Female | |
| 1 | 3 | 3 | | 0 | | 0 | |
| 2 | 3 | 3 | | 1 | | 0 | |
| 3 | 3 | 3 | | 2 | | 0 | |
| 4 | 3 | 1 | | 1 | | 0 | |
| 5 | 3 | 3 | | 1 | | 0 | |
| 6 | 2 | 0 | | 0 | | 1 | |
| 7 | 1 | 3 | | 3 | | 1 | |
| 8 | 3 | 3 | | 0 | | 0 | |
| 9 | 3 | 3 | | 1 | | 1 | |
| 10 | 3 | 3 | | 1 | | 0 | |
| 11 | 1 | 3 | | 1 | | 0 | |
| 12 | 3 | 3 | | 1 | | 0 | |
| 13 | 0 | - | | 0 | | 1 | |
| 14 | 3 | - | | - | | 1 | |
| 15 | 0 | - | | - | | 0 | |
| 16 | 3 | - | | - | | 0 | |
| 17 | 3 | - | | - | | 0 | |
| 18 | - | - | | - | | 0 | |
| 19 | - | - | | - | | 0 | |
| # samples: | 17 | 12 | | 13 | | 19 | |
| average: | **2.35** | **2.58** | | **0.92** | | **0.26** | |
| SEM: | **0.27** | **0.29** | | **0.24** | | **0.10** | |
| SD: | 1.11 | 1.00 | | 0.86 | | 0.45 | |
|  |  |  | |  | |  | |
| *Extent of branching in the labyrinth (0= absent, 1= initiated, 2= intermediate, 3= normal) | | | | | | | |
