# Supplementary material for: Loss of DNMT1o Disrupts Imprinted X Chromosome Inactivation and Accentuates Placental Defects in Females
Source: PLoS Genet. 2013 Nov 21;9(11):e1003873. doi: 10.1371/journal.pgen.1003873 (PMC3836718; doi:10.1371/journal.pgen.1003873)
Supplement: Table S5 — (related to Figure S2B). Trophoblast giant cells per section in 9.5dpc extraembryonic tissues from litters of control and Dnmt1omat−/− females. (DOCX) [file pgen.1003873.s010.docx]

| **Table S5 (related to Figure S2B)**. Trophoblast giant cells per section in 9.5dpc extraembryonic tissues from litters of control and *Dnmt1o^mat-/-^* females. | | | | |
| --- | --- | --- | --- | --- |
|  | | | | |
|  | Trophoblast Giant Cells | | | |
|  | Control | | *Dnmt1o^mat-/-^* | |
| Placenta | Male | Female | Male | Female |
| 1 | 84 | 152 | 164 | 125 |
| 2 | 95 | 91 | 131 | 101 |
| 3 | 135 | 119 | 124 | 212 |
| 4 | 97 | 131 | 110 | 177 |
| 5 | 153 | 114 | 74 | 149 |
| 6 | 91 | 114 | 157 | 293 |
| 7 | 110 | 22 | 157 | 142 |
| 8 | 100 | 117 | 69 | 194 |
| 9 | 109 | 87 | 116 | 184 |
| 10 | 85 | 84 | 133 | 149 |
| 11 | 101 | 72 | 99 | 125 |
| 12 | 142 | 114 | 168 | 50 |
| 13 | 145 | - | 178 | 194 |
| 14 | 82 | - | - | 176 |
| 15 | 108 | - | - | 132 |
| 16 | 84 | - | - | 144 |
| 17 | - | - | - | 181 |
| 18 | - | - | - | 235 |
| 19 | - | - | - | 151 |
| # samples: | 16 | 12 | 13 | 19 |
| average: | **108** | **101** | **129** | **164** |
| SEM: | **6** | **10** | **10** | **12** |
| SD: | 24 | 33 | 35 | 52 |
