# Supplementary material for: Loss of DNMT1o Disrupts Imprinted X Chromosome Inactivation and Accentuates Placental Defects in Females
Source: PLoS Genet. 2013 Nov 21;9(11):e1003873. doi: 10.1371/journal.pgen.1003873 (PMC3836718; doi:10.1371/journal.pgen.1003873)
Supplement: Table S6 — (related to Table 1). Unidentified loci that display altered DNA methylation in Dnmt1omat−/− embryos and placentas. (DOCX) [file pgen.1003873.s011.docx]

| **Table S6 (related to Table 1).** Unidentified loci that display altered DNA *methylation in Dnmt1o^mat-/^****^-^***embryos and placentas. | | | | | | | | | | |  |  |
| --- | --- | --- | --- | --- | --- | --- | --- | --- | --- | --- | --- | --- |
|  | |  |  | |  | |  | |  | |  |  |
| **Placenta *Dnmt1o^mat-/-^*** | | |  | |  | |  | |  | |  |  |
|  |  | | **Meth. change^a^** | | | | **Methylation (%)** | | | |  |  |
| **RLGS Spots** |  | | **XX** | | **XY** | | **Control** | | ***Dnmt1o^mat-/-^*** | |  |  |
| 2G116 | hypomethylation (1/8) | | 1 | | 0 | | 100 | | 25 | |  |  |
| 1F61 | hypomethylation (1/8) | | 1 | | 0 | | 100 | | 0 | |  |  |
| 1D19 | hypomethylation (3/8) | | 3 | | 0 | | 100 | | 25-90 | |  |  |
| 5C11 | hypomethylation (4/8) | | 4 | | 0 | | 50-75 | | 25 | | all-XX**^d^** |  |
| 2C66 | hypomethylation (4/8) | | 4 | | 0 | | 50-75 | | 0-25 | | all-XX**^d^** |  |
| 3F92 | hypomethylation (4/8) | | 4 | | 0 | | 100 | | 25-50 | | all-XX**^d^** |  |
| 2E56 | hypomethylation (4/8) | | 4 | | 0 | | 50 | | 25 | | all-XX**^d^** |  |
| 3E26 | hypomethylation (4/8) | | 4 | | 0 | | 25 | | 0 | | all-XX**^d^** |  |
| 2B52 | hypomethylation (5/8) | | 4 | | 1 | | 90-100 | | 50-75 | |  |  |
| 2D60 | hypomethylation (5/8) | | 3 | | 2 | | 75 | | 25-50 | |  |  |
| 4D01 | hypomethylation (6/8) | | 4 | | 2 | | 90 | | 50-75 | |  |  |
| 3E86 | hypomethylation (6/8) | | 4 | | 2 | | 100 | | 50-75 | |  |  |
| 1D27 | hypomethylation (7/8) | | 4 | | 3 | | 25 | | 0-10 | |  |  |
| 4F102 | hypomethylation (7/8) | | 4 | | 3 | | 100 | | 50-90 | |  |  |
| 3F93 | hypomethylation (8/8) | | 4 | | 4 | | 90 | | 50-75 | | all**^c^** |  |
| 2G107 | hypomethylation (8/8) | | 4 | | 4 | | 100 | | 50-75 | | all**^c^** |  |
| 1F41 | hypermethylation (1/8) | | 0 | | 1 | | 0**^b^** | | 25 | |  |  |
| 3E26 | hypermethylation (1/8) | | 0 | | 1 | | 25 | | 50 | |  |  |
| 5C11 | hypermethylation (2/8) | | 0 | | 2 | | 50-75 | | 90 | |  |  |
| 2G-39 | hypermethylation (5/8) | | 1 | | 4 | | 25 | | 50-62 | |  |  |
| 2E34 | hypermethylation (6/8) | | 3 | | 3 | | 10 | | 25-50 | |  |  |
| 3E20 | hypermethylation (8/8) | | 4 | | 4 | | 0 | | 25-50 | | all**^c^** |  |
|  |  | |  | |  | |  | |  | |  |  |
| **^a^** Number of profiles with methylation change for specific loci (total samples 4XX + 4XY) | | | | | | | | | | |  |  |
| **^b^** RLGS spot intensity suggests that methylation was even more reduced then intensity normally attributed for 0% methylation. | | | | | | | | | | | |  |
| **^c^** Loci methylation changed in all profiles | | | |  | |  | |  | |  | |  |
| **^d^** Methylation changed in all XX profiles and unchanged in all XY profiles | | | | | | | | | |  | |  |
