# Supplementary material for: Loss of DNMT1o Disrupts Imprinted X Chromosome Inactivation and Accentuates Placental Defects in Females
Source: PLoS Genet. 2013 Nov 21;9(11):e1003873. doi: 10.1371/journal.pgen.1003873 (PMC3836718; doi:10.1371/journal.pgen.1003873)
Supplement: Table S7 — List of primer sets and sequences used in PCR based experiments. (DOCX) [file pgen.1003873.s012.docx]

| **Table S7**. List of primer sets and sequences used in PCR based experiments. | | | | |  |
| --- | --- | --- | --- | --- | --- |
| **Target Regions** | **Forward (5'-3')** | | **Reverse (5'-3')** | |  |
| **Sequenom MassArray** | | | | |  |
| *MeCP2*_2 | aggaagagagTGTGGGATTTAGGAATATAATGTTTAGT | | cagtaatacgactcactatagggagaaggctTCCTACCCTTCTTAAAACTTTAAACC | |  |
| *Chic1*_1 | aggaagagagTGATGAGTATTTTGTTGTTTAATATGG | | cagtaatacgactcactatagggagaaggctTTTTAACCAAATACCCTTTACTAAAAAT | |  |
| *Chic1*_2 | aggaagagagTTATGTTGGGTAGTAGGATGTTTATT | | cagtaatacgactcactatagggagaaggctTTTTAAACAATATCCTAAACTCTCTTTT | |  |
| *Atp7a*_1 | aggaagagagGGTTTATGATATTTTATGTTGGTAAGGAT | | cagtaatacgactcactatagggagaaggctAAAACTCACCTAAATTTCCAAAACT | |  |
| *Pgk1*_2 | aggaagagagTTTTTGGGTTTAGAGGTTGGG | | cagtaatacgactcactatagggagaaggctTCCAACTTATCCAAAATCAACTTAT | |  |
| *Hprt1*_1 | aggaagagagGGATTTTTGGAGTTTGGTTAGATGT | | cagtaatacgactcactatagggagaaggctAATCCAAACCTAAACATACCCTCTC | |  |
| *Hprt1*_2 | aggaagagagTGGGGTATTTAGTTAGATTTTAGGAAT | | cagtaatacgactcactatagggagaaggctTCAAACCCACCTAATCAAATAAAAA | |  |
| *Abc7*_1 | aggaagagagTGGGTAATTTTGTATTTTATTTGGT | | cagtaatacgactcactatagggagaaggctAAATTCCCCTATCCTTCACCCTACT | |  |
| *Rnf12*_1 | aggaagagagTTGTTAGTGTTAGGTAATAATGGGGA | | cagtaatacgactcactatagggagaaggctACCCCTACCTACCTCCAATCC | |  |
| *Jarid1c*_1 | aggaagagagGGAATAGGATTAAGAATTTGTAAAAGG | | cagtaatacgactcactatagggagaaggctTAAAACATCCACAAAATACCAACCA | |  |
| *Jarid1c*_2 | aggaagagagTTGTGTATTGAATTAAGGGAAATTTTTA | | cagtaatacgactcactatagggagaaggctAACCCACCATAAAACTAAAATCC | |  |
| *Ogt*_2 | aggaagagagGTGATGGTTAAGAGAATTTTTGGAA | | cagtaatacgactcactatagggagaaggctCCCATCAATATTATAACCTTCTCTCTC | |  |
| *Acsl4*_2 | aggaagagagTATAGGGAAGAGTTAGGTGGGGTTT | | cagtaatacgactcactatagggagaaggctCCTTACCAAACAACTCCAAAACTAA | |  |
| *Brwd3*_1 | aggaagagagTTTTTGGGTTAGAATATTTATGGGT | | cagtaatacgactcactatagggagaaggctTTACAAAATCCAAACTACAAAAACCT | |  |
| *GM784*_2 | aggaagagagGGTTAATAGGTTAGGGGTTGGATGT | | cagtaatacgactcactatagggagaaggctCAATCACAAATACAAAAACAAACAAA | |  |
| *02M06Rik*_2 | aggaagagagTTAAGATGTTATTAGGGTTTTGGGG | | cagtaatacgactcactatagggagaaggctAATCCTAACACCATTCCCTATCAAT | |  |
| *Pak3*_1 | aggaagagagGGAATAGTTAGGTTTGGGAGATTT | | cagtaatacgactcactatagggagaaggctTCCTCCCAATAACTCTAAAAACCAC | |  |
| *Smarca1*_1 | aggaagagagGTTAAAAGGTTAGGGTTTGGGTTTT | | cagtaatacgactcactatagggagaaggctATATTCTCCACCCCTACTCTAACAAA | |  |
| *Zic3*_1 | aggaagagagAGAGTAGGGTTAAGTTAGGGAGAGG | | cagtaatacgactcactatagggagaaggctAACTAACCTAAAATCACCTAACCCC | |  |
| *Zic3*_2 | aggaagagagGTTTAGGGGGTTTTTAGGTAGTG | | cagtaatacgactcactatagggagaaggctAATAACCCAAAACATTAACATAACC | |  |
| *Zic3*_3 | aggaagagagTTGATTTTTATTAATAGGGTTTTTGG | | cagtaatacgactcactatagggagaaggctACCCCTTTTAAATCAACCTCATACT | |  |
| *Rbbp7*_1 | aggaagagagAGTAGGTTTGTGGGTTGTTGTTGT | | cagtaatacgactcactatagggagaaggctTTAAATCATTAATTACCCTACCACC | |  |
| *Rbbp7*_2 | aggaagagagTGGAGAAAGTTTATAATTAAGTGGTTGA | | cagtaatacgactcactatagggagaaggctAAAAACAAACCTATAAACTACTACTATTCC | |  |
| *Hccs*_1 | aggaagagagTTGAAGTTTATGTTATTATTTGGGGA | | cagtaatacgactcactatagggagaaggctAAAAACAACACTAATAACTTCCTACCC | |  |
| *Lonrf3*_1 | aggaagagagTTTAGGAATAGGGAATTAGTAGGAAAGA | | cagtaatacgactcactatagggagaaggctAAATTATATACCTCTCCCCAATCCA | |  |
| *Lonrf3*_2 | aggaagagagTGAGTAGAGTTGTTTTTATTTGGTG | | cagtaatacgactcactatagggagaaggctCCACACCTTTTATAAACTATACCTAAAAC | |  |
| *Klhl13*_1 | aggaagagagAGTTTGGAATAGGGATTTTGGTAAA | | cagtaatacgactcactatagggagaaggctAAAAACCTAAAAATACCCAAACTTC | |  |
| *Klhl13*_2 | aggaagagagTTGGTTTAGTTTTTAGGTAGTGGGA | | cagtaatacgactcactatagggagaaggctAAACTCCAAAACCACTTAACAATCA | |  |
| *Xist* | aggaagagagTGTTGTGTGAGTGAATTTATGGTTT | | cagtaatacgactcactatagggagaaggctTAAACCCTATCCCCTAATCCTCTAC | |  |
| *Tsix*-CTCFc* | aggaagagagTTTAAAGGTAAATGGGTTTAGGTTG | | cagtaatacgactcactatagggagaaggctAAACACTTTAAACATCTACCCTCTCC | |  |
| *Tsix*-CGI | aggaagagagTGGAGTTATTTTAGTATATTTGGTAAAGG | | cagtaatacgactcactatagggagaaggctACAAAAACCCTCTATACCATCCAAA | |  |
| *Tsix*-upstream | aggaagagagGGTTAAGTGTTTGTAGGATAGTGGG | | cagtaatacgactcactatagggagaaggctTTAATAAAATCTCCCAATCCCAAAC | |  |
| *Xite*-DHS4* | aggaagagagTAGATTGTTATGGGGGTGTTTTAGA | | cagtaatacgactcactatagggagaaggctAACCCTCTAATAAATCCATCAACCT | |  |
| *Xite*-DHS2* | aggaagagagGTATGTTGAATTATTTTTGTATTTTATGAG | | cagtaatacgactcactatagggagaaggctATCTCTAAATCACCACCACCTACAA | |  |
| *Xite*-DHS6* | aggaagagagTTGGATGAATTGGAATTTATATTTTAGA | | cagtaatacgactcactatagggagaaggctTCCTCCCTATCACTACTACCATTAAAC | |  |
| *U2af1*-rs1 | aggaagagagTGGTGGGGTTTTTAGAGTTTTTAGT | | cagtaatacgactcactatagggagaaggctATACCACCCTAAAATTACTATCCAAAA | |  |
| *Snrpn*_1 | aggaagagagGGGTTTTAGGATGTAGGAGTTTTGT | | cagtaatacgactcactatagggagaaggctAACTCCCCCAAACATAAACTAAAAA | |  |
| *Snrpn*_2 | aggaagagagTTTAAAAATAAAGGTATTTGGGTTGTT | | cagtaatacgactcactatagggagaaggctTCAAACATTCCTTTTAATAACTACCTTT | |  |
| *Igf2r*_1 | aggaagagagAGGAGTTAAAGTTTGGTGAGGTTG | | cagtaatacgactcactatagggagaaggctCCCTCCTATAAAACCCTTCCTTTAC | |  |
| *Igf2r*_2 | aggaagagagGGTTAAGGGTGAAAAGTTGTATAAGG | | cagtaatacgactcactatagggagaaggctCTTAACATAACCAAAATCACAACCC | |  |
| *H19*_1 | aggaagagagGTTGATGGTTTTAGAATTTTATAAGTTAG | | cagtaatacgactcactatagggagaaggctCACAAATACCACTAAAAAAACAAAACA | |  |
| *H19*_2 | aggaagagagTTGTGAATTTTAATATTAGGGGTGG | | cagtaatacgactcactatagggagaaggctACAAAACACTTACACCCAAAACTCA | |  |
| *H19*_3 | aggaagagagGGTTTTATGAAGTTTATGATTATGGGA | | cagtaatacgactcactatagggagaaggctAAAAATTCTACAAAAAAACCATACCC | |  |
| IAP_1 | aggaagagagGTGAGTAATTGTTATTATAAGATGG | | cagtaatacgactcactatagggagaaggctTTACTCACCCATCACCCCA | |  |
| GSAT_1 | aggaagagagTGAGAAATGTATATTGAAAGATTTGGA | | cagtaatacgactcactatagggagaaggctAAACATTTCTAAATTTTCCACCTTTT | |  |
| LINE1_1 | aggaagagagGGGGAATAAAATATTTATGGAAGGA | | cagtaatacgactcactatagggagaaggctCCAAACACTAACATAACCTCACAAA | |  |
| LINE1_2 | aggaagagagGGGTTTTTAATGAAGGAGTTAGAGAA | | cagtaatacgactcactatagggagaaggctCCCTAACATTCCCCTATACTAAAACA | |  |
| B1-SINE_1 | aggaagagagGTATTTGGGAGGTAGAGGTAGG | | cagtaatacgactcactatagggagaaggctTCCTAAAACTCACTTTATAAACCAAA | |  |
| **Sexing** | | | | |  |
| *Myog* | TTACGTCCATCGTGGACAGC | | TGGGCTGGGTGTTAGTCTTA | |  |
| *Zfy1/2* | AAGATAAGCTTACATAATCACATGGA | | CCTATGAAATCCTTTGCTGCACATGT | |  |
| *Gapdh* | ATGTTTGTGATGGGTGTGAA | | TCCTCAGTGTAGCCCAAGAT | |  |
| *Xist* | TTGCGGGATTCGCCTTGAT | | TGAGCAGCCCTTAAAGCCAC | |  |
| **X-Chromosome Allele Specific Expression (Restriction Enzymes)** | | | | |  |
| *Mecp2* (**DdeI**) | CATGGTAGCTGGGATGTTAGG | | GCAATCAATTCTACTTTAGAGCG | |  |
| *Ogt* (**AvaI**) | TCTCGAGTTGCAGCTTCTCA | | CATGTGGTCAGGTTTGTTGC | |  |
| *Tsix* (**BsmaI**) | TGGGTCATTGGCATCTTAGTC | | CCCAGGGTGTCTGATCTCTT | |  |
| *Rlim* (**HaeIII**) | GAGCCCCGATGAAAATAGAGC | | GGTCGGCACTTCTGTTACTGC | |  |
| *Abc7*(**NlaIII**) | AAGCATTCGGCAGTTCTGACC | | TCTAGTATCAACATCCTTTAACCC | |  |
| *Jarid1c* (**NspI**) | CCTGGCAGCAGCTGTACATA | | TAGCCCAGGTGTCAAAGGAC | |  |
| **Bisulfite Sequencing** | | | | |  |
| *Chic1-*outside | GGGAATAGATTGAAGAGAAAGAGG | | TTTTAAACAATATCCTAAACTCTCTTTT | |  |
| *Chic1-*inside | GGGAATAGATTGAAGAGAAAGAGG | | CCTCAACTATCTACATAAAAACAAATATAT | |  |
| *Xite*-outside | TTGGATGAATTGGAATTTATATTTTAGA | | TCCTCCCTATCACTACTACCATTAAAC | |  |
| *Xite*-inside | TTGTTTTTAAATGTTGGGATTAAAG | | TCCTCCCTATCACTACTACCATTAAAC | |  |
| * Numencleature from Boumil et al. 2006 | |  | |  | |
