# Supplementary material for: Loss of DNMT1o Disrupts Imprinted X Chromosome Inactivation and Accentuates Placental Defects in Females
Source: PLoS Genet. 2013 Nov 21;9(11):e1003873. doi: 10.1371/journal.pgen.1003873 (PMC3836718; doi:10.1371/journal.pgen.1003873)
Supplement: Text S1 — Supporting Results details. DNMT1o deficiency is associated with sex-specific abnormalities in extraembryonic tissues. (DOCX) [file pgen.1003873.s013.docx]

**Supporting Results Details**

**DNMT1o deficiency is associated with sex-specific abnormalities in extraembryonic tissues**

The macroscopic extraembryonic defects associated with maternal DNMT1o deficiency correlated with histomorphological abnormalities. As shown in Figure S2A sections i&ii, in *Dnmt1o^mat+/-^* (control) and histologically normal male *Dnmt1o^mat-/-^* extraembryonic tissues, chorioallantoic attachment was apparent and the emerging labyrinth formed a dense network of villous structures (for review see [S1]). However, detailed histological examination showed that cellular morphology in the *Dnmt1o^mat-/-^* extraembryonic tissue was defective. Improper folding and interdigitation of the chorionic layer to form the primary villi of the labyrinth (arrows, Figure S2A sections i&ii) was observed for the vast majority of *Dnmt1o^mat-/-^* conceptuses (Figure S2A sections iii&iv, Figure S2B, Table S4). Although the allantois remained tightly associated with the chorionic plate, the resulting branching pattern to create villi was significantly impaired in *Dnmt1o^mat-/-^* versus control tissues (p<0.05) resulting in an extremely compact chorionic plate. Failure to generate a normal branching pattern was more severe in female *Dnmt1o^mat-/-^* versus male tissues (p<0.01). In most *Dnmt1o^mat-/-^* females, initiation of branching patterns was impeded (Table S4) resulting in a very dense chorionic plate (Figure S2A-iii). In addition to the faulty interdigitation process associated with a lack of DNMT1o, we also observed an expanded population of giant cells (Figure S2A sections iii&iv, Figure S2B and Table S5). Normally, a single layer of giant cells separates the outer surface of the placenta from the maternal decidual cells (Figure S2A section i). Giant cell hyperproliferation was influenced in a sex-specific manner by *Dnmt1o^mat-/-^*; thus, only the extra-embryonic tissues of the *Dnmt1o^mat-/-^* females revealed a significant increase in total cell numbers (164 vs. 129, p<0.05) (Figure S2B). The substantial increase in the giant cell population caused an overgrowth of this sheet-like layer. Taken together, while both males and females showed evidence of extra-embryonic abnormalities, we also detected significant female-specific impairment of extraembryonic tissue organization in *Dnmt1o^mat-/-^* conceptuses.

**Supporting References**

S1. Watson ED, Cross JC (2005) Development of structures and transport functions in the mouse placenta. Physiology (Bethesda) 20: 180-193.
