# Supplementary material for: Loss of DNMT1o Disrupts Imprinted X Chromosome Inactivation and Accentuates Placental Defects in Females
Source: PLoS Genet. 2013 Nov 21;9(11):e1003873. doi: 10.1371/journal.pgen.1003873 (PMC3836718; doi:10.1371/journal.pgen.1003873)
Supplement: Text S2 — Supporting Discussion details. Relationship between global B1-SINE methylation and the Xic. (DOCX) [file pgen.1003873.s014.docx]

**Supporting Discussion Details**

**Relationship between global B1-SINE methylation and the *Xic***

Extraembryonic tissues from *Dnmt1o^mat-/-^* conceptuses and XX ESCs represent two unique situations where two active X chromosomes are maintained for an unnaturally prolonged period. Both of these conditions result in a global reduction of repeat methylation. Interestingly, wild-type and *Dnmt1o^mat-/-^* females displayed a lower level of methylation compared to males of the same *Dnmt1* genotypes for B1-SINE in both placenta and embryo (p<0.00001), a pattern observed for only this repeat. In support of this, others have found lower methylation levels in Alu repeats (equivalent to the mouse B1-SINE) in women [S[2](#_ENREF_1)]. The specific behavior of this repeat class may have to do with the unique genomic distribution of Alu sequences in the genome compared to other common repetitive elements [S[3](#_ENREF_2)]. B1/Alu sequences are enriched in genic regions, whereas other common repeat families, including GSAT, IAP and LINE1, are enriched in gene-poor, heterochromatic regions. Indeed, we observed that the repeats that are more commonly found in heterochromatic regions - IAP, LINE1 and GSAT - exhibit a high level of coordinated methylation within samples, whereas B1-SINEs have a distinct pattern (Figure 6D). The *Xite* locus is rich in B1-SINE repeats; the *Xite*-DHS6 genomic sequence measured here partly contains a B1-SINE repeat (although there are no CpGs within the assayed portion of this B1 element). The observation of a strong positive correlation between the B1-SINE-rich *Xite*-DHS6 locus and global B1-SINE methylation suggests the possibility of a functional relationship between the *Xic* and regulation of global repeat methylation.

**Supporting References**

S2. El-Maarri O, Becker T, Junen J, Manzoor SS, Diaz-Lacava A, et al. (2007) Gender specific differences in levels of DNA methylation at selected loci from human total blood: a tendency toward higher methylation levels in males. Hum Genet 122: 505-514.

S3. Tsirigos A, Rigoutsos I (2009) Alu and b1 repeats have been selectively retained in the upstream and intronic regions of genes of specific functional classes. PLoS Comput Biol 5: e1000610.
