# Supplementary material for: Loss of DNMT1o Disrupts Imprinted X Chromosome Inactivation and Accentuates Placental Defects in Females
Source: PLoS Genet. 2013 Nov 21;9(11):e1003873. doi: 10.1371/journal.pgen.1003873 (PMC3836718; doi:10.1371/journal.pgen.1003873)
Supplement: Text S3 — Supporting Materials and Methods. (DOCX) [file pgen.1003873.s015.docx]

**Supporting Materials and Methods**

**Animals**

The mutant *Dnmt1o^-/-^* was maintained in the inbred 129/Sv background and mice were genotyped using a PCR assay as previously described [1[2](#_ENREF_1)]. All animal procedures were carried out in accordance with the Canadian Council of Animal Care.

**Assessment of extraembryonic hyperplasia and histological analysis.**

Homozygous DNMT1o-deficient females (*Dnmt1o^mat-/-^*) or heterozygous *Dnmt1o^mat+/-^* females were crossed to wild-type 129/Sv males (Charles River Canada, St-Constant, Qc. Canada). The presence of a vaginal plug was designated 0.5 days post-coitum (dpc). Embryos and placentae were collected from the uteri at 9.5dpc and extraembryonic hyperplasia was assessed by measuring the excised placenta flat under the microscope. For the histological analysis, implantation sites were collected at 9.5dpc, fixed in 10% formaldehyde overnight at 4^o^C and embedded in paraffin. Five μM thick sagittal serial sections were cut and every 20^th^ section was stained with hematoxylin/eosin (H&E). The site of umbilical attachment was always used as the reference point for comparison between sections from mutants and controls. The gender of the embryo was assessed by PCR on DNA extracted from either fresh yolk sac tissue using the QIAamp DNA Micro Kit (Qiagen, Mississauga, Ont. Canada) or from the sectioned material using a 30G gauge needle to scrape cells of the sections and then using the QIAamp DNA FFPE tissue kit version (Qiagen). The male specific *Zfy1* primers generated a 363-bp product and *Myog*, used as an internal control, amplified a 245-bp region (Table S7).

**DNA methylation analyses**

Quantitative DNA methylation analyses were accomplished using the EpiTYPER application (Sequenom, San Diego, Ca.) as previously described [60]. Briefly, genomic DNA was isolated from 9.5dpc wild type and *Dnmt1o^mat-/-^* mice embryos and placentae using the QIAamp DNA mini Kit (Qiagen) and subjected to bisulfite treatment. Candidate regions of imprinted DMDs, X chromosome-CGI and repeat elements were amplified using primers (Table S7) designed for bisulfite treated DNA. Amplified DNA was then transcribed in vitro and and products cleaved using RNAse A. The molecular weight of the resulting cleavage products indicative of the DNA methylation state was analyzed using matrix-assisted laser desorption ionization time-of-flight (MALDI-TOF) mass spectrometry. DNA methylation standards (0, 20, 40, 60, 80 and 100% methylation at CpG dinucleotides) were used to control for PCR amplification bias. Equation fitting algorithms based on the R statistical computing environment were used for data correction. Display of methylation results as heat maps and unsupervised clustering were performed using the Multiple Experimental Viewer software (http://www.tm4.org/mev.html).

Restriction landmark genomic scanning (RLGS) was used to assess genome-wide methylation in 9.5dpc control (*Dnmt1o^mat+/-^*) and *Dnmt1o^mat-/-^* mice placentae. *Dnmt1o^mat+/-^* and *Dnmt1o^mat-/-^* conceptuses had identical 129/Sv genetic backgrounds to control for any strain-specific effects in methylation and/or RLGS spot location. Sufficient DNA was obtained from single control and single *Dnmt1o^mat-/-^* 9.5dpc placentae, however in the case of smaller samples, pools of similar placentae (same sex and similar morphology) were used to obtain sufficient tissue for DNA extraction and RLGS studies. High-molecular weight DNA was isolated using proteinase K digestion and phenol-chloroform extraction, and RLGS was performed on the extracted DNA as described [[33](#_ENREF_3)]. RLGS gels were dried and exposed to autoradiographic film (GE Healthcare/Amersham) and phosphor imaging. Each spot was analyzed and intensities were compared. Methylation scores ranged from 0% methylation (intensively dark spot) to 100% methylation (absence of spot). Spot identities were determined by using the virtual RLGS method [[34](#_ENREF_4)] and confirmed by obtaining bacterial artificial chromosome clones (Roswell Park Microarray Core Facility, Buffalo, NY) and running RLGS mixing gels as previously described [S4].

**RNA and DNA FISH**

The *Xist* probe used was pEFB [66, S5] and the X chromosome probe was DXwas70 [[67](#_ENREF_7)]. Preimplantation embryos were flushed from the uterine horns and attached to poly-lysine treated superfrosted slides, exposed to an acidic tyrode solution, washed in cytoskeletal (CSK) buffer, fixed in 4% paraformaldehyde and keep at -20^o^C in 70% ethanol until FISH experiments. RNA FISH and washes were performed as described [[65](#_ENREF_8)] using the pEFB probe labeled with Spectrum Green dUTP (Vysis, Downer Grove, IL). Images were obtained with an Olympus epifluorescent microscope. The slides were then processed for DNA FISH as previously described by Duthie et *al*., [[S5](#_ENREF_9)]. The DXwas70 probe was labeled with Spectrum Red dUTP (Vysis, Downer Grove, IL). Further images were obtained of the DNA FISH signals as described above.

**Allele-specific expression assay by RT-PCR**

To separate the parenthood of the active and inactive X chromosome alleles in female *Dnmt1o^mat-/-^* extraembryonic tissues, we generated 9.5 dpc conceptuses from inbred 129/Sv mothers (*Dnmt1o^mat-/-^* and control) and CAST/EiJ fathers to generate single nucleotide polymorphisms (SNPs) along the X chromosome. The visceral endoderm layer of yolk sacs from the conceptuses was collected followed by RNA isolation (RNeasy; Qiagen) and on column DNase treatment (Qiagen). Reverse transcription was performed with random primers and Superscript II (Invitrogen, Burlington, Ont. Canada) following the manufacturer’s recommendations. X chromosome linked genes containing polymorphisms were amplified with the use of flanking primers (Table S7) in RT-PCR. RT-PCR products were digested with proper restriction enzymes to reveal polymorphic insertions and fractionated on acrylamide gels (8 or 10%). Gels were soaked in Sybr gold (Invitrogen) for 15 min and finally photographed under UV illumination.

**Supporting References**

S4. Smiraglia DJ, Fruhwald MC, Costello JF, McCormick SP, Dai Z, et al. (1999) A new tool for the rapid cloning of amplified and hypermethylated human DNA sequences from restriction landmark genome scanning gels. Genomics 58: 254-262.

S5. Duthie SM, Nesterova TB, Formstone EJ, Keohane AM, Turner BM, et al. (1999) Xist RNA exhibits a banded localization on the inactive X chromosome and is excluded from autosomal material in cis. Hum Mol Genet 8: 195-204.
